# Differential cellular responses to adhesive interactions with galectin-8- and fibronectin-coated substrates

**DOI:** 10.1242/jcs.252221

**Published:** 2021-04-27

**Authors:** Wenhong Li, Ana Sancho, Wen-Lu Chung, Yaron Vinik, Jürgen Groll, Yehiel Zick, Ohad Medalia, Alexander D. Bershadsky, Benjamin Geiger

**Affiliations:** 1 Department of Immunology, Weizmann Institute of Science, Rehovot, 7610001, Israel; 2 Department of Functional Materials in Medicine and Dentistry and Bavarian Polymer Institute, University of Würzburg, Würzburg, 97070, Germany; 3 Department of Automatic Control and Systems Engineering, University of the Basque Country UPV/EHU, San Sebastian, 20018, Spain; 4 Department of Biochemistry, University of Zurich, Zurich, CH-8057, Switzerland; 5 Department of Molecular Cell Biology, Weizmann Institute of Science, Rehovot, 7610001, Israel; 6 Mechanobiology Institute, National University of Singapore, 117411 Singapore

**Keywords:** Extracellular matrix, Focal adhesions, Filopodia, Lamellipodia, Myosin-II, Rho GTPases

## Abstract

The mechanisms underlying the cellular response to extracellular matrices (ECMs) that consist of multiple adhesive ligands are still poorly understood. Here, we address this topic by monitoring specific cellular responses to two different extracellular adhesion molecules – the main integrin ligand fibronectin and galectin-8, a lectin that binds β-galactoside residues  − as well as to mixtures of the two proteins. Compared with cell spreading on fibronectin, cell spreading on galectin-8-coated substrates resulted in increased projected cell area, more-pronounced extension of filopodia and, yet, the inability to form focal adhesions and stress fibers. These differences can be partially reversed by experimental manipulations of small G-proteins of the Rho family and their downstream targets, such as formins, the Arp2/3 complex and Rho kinase. We also show that the physical adhesion of cells to galectin-8 was stronger than adhesion to fibronectin. Notably, galectin-8 and fibronectin differently regulate cell spreading and focal adhesion formation, yet act synergistically to upregulate the number and length of filopodia. The physiological significance of the coherent cellular response to a molecularly complex matrix is discussed.

This article has an associated First Person interview with the first author of the paper.

## Abstract (precis)

**Summary:** Fibronectin and galectin-8 have dramatically different effects on cell spreading and focal adhesion formation; however, when mixed, they synergize in stimulating filopodia formation.

## INTRODUCTION

In multicellular organisms, the majority of cells interact with the extracellular matrix (ECM), a multi-molecular network comprising the cells' microenvironment (Cárcamo et al., 2006; Diskin et al., 2012). ECM components are synthesized, secreted and assembled by cells, and form specialized tissue scaffolds, characterized by specific biochemical, topographical and mechanical features. ECM networks serve as primary sources of environmental information for many cell types, normal and transformed, affecting their shape, adhesive properties, cytoskeletal organization and migration (Bonnans et al., 2014; Humphrey et al., 2014; Muncie and Weaver, 2018).

Several classes of cell surface receptor interact with the ECM, and convey to cells its biochemical and mechanical characteristics. Among these are transmembrane receptors of the integrin family and diverse proteoglycans (Humphries et al., 2019; Multhaupt et al., 2016). Depending on the type of matrix and the specific cellular context, matrix receptors assemble into different types of adhesion complexes through which they interact with the cytoskeleton. Adhesion complexes, such as focal adhesions (Geiger et al., 2009), podosomes (Alonso et al., 2019; Schachtner et al., 2013), hemidesmosomes (Walko et al., 2015), filopodia (Jacquemet et al., 2015) and adhesion waves (Case and Waterman, 2011) are formed by several hundreds of structural and signaling proteins, which collectively mediate the adhesive and signaling functions of these structures.

Thus far, our knowledge and understanding of the processes that underlie matrix-dependent signaling is based on the use of specific matrix proteins, such as fibronectin, collagen or laminins, presented to cells on substrates of variable topographies and rigidities. Such studies indicated that even under similar topographical and mechanical conditions, different matrix proteins can generate distinct and, sometimes, contradictory cellular responses (Adams et al., 2001; Adams and Schwartz, 2000; Midwood et al., 2016; Midwood and Schwarzbauer, 2002; Resovi et al., 2014; Wenk et al., 2000). Although the phenomenon of ECM functional diversity is widely appreciated, the downstream signaling pathways and even the basic phenotypic manifestations are poorly characterized.

Galectins comprise a large family of adhesive animal lectins, are expressed in multiple tissues and display diverse functions, including the regulation of cell adhesion and migration. They are secreted by cells through non-conventional mechanisms, bypassing the endoplasmic reticulum- and/or Golgi-dependent secretary pathway (Popa et al., 2018), and function as matricellular proteins serving as soluble ligands crosslinking molecules on the cell surface, as well as components of the extracellular matrix (Elola et al., 2007; He and Baum, 2006; Nabi et al., 2015). All galectins bind to β-galactoside and interact with membrane glycoproteins and glycolipids. Depending on the organization of sugar moieties and protein structures, different galectins can interact with a variety of molecules on the cell surface (Johannes et al., 2018).

A prominent member of the galectin family is galectin-8, a molecule that contains two carbohydrate-recognition domains (CRDs), connected by a linker of variable lengths, and of which three different isoforms (Gal-8S, Gal-8M, Gal-8L) have been identified (Troncoso et al., 2014, Hadari et al., 1997; Zick et al., 2002). Galectin-8 plays an important role in normal physiological processes, such as vascular and lymphatic angiogenesis (Troncoso et al., 2014), platelet activation (Romaniuk et al., 2015, 2010), polarization of T-lymphocytes (Cárcamo et al., 2006), and limb development (Newman et al., 2018). At the same time, galectin-8 is often overexpressed and secreted by some types of tumor cells (Elola et al., 2014; Vinik et al., 2018), a phenomenon thought to be crucial to their metastatic ability (Gentilini et al., 2017; Zick et al., 2002).

When immobilized on a rigid substrate, galectin-8 can support the adhesion and spreading of several cell types (Cárcamo et al., 2006; Diskin et al., 2012; Levy et al., 2001), initiates downstream signaling via tyrosine phosphorylation of focal adhesion proteins (Diskin et al., 2012; Levy et al., 2001), and activation of Rho and Rac1 (Cárcamo et al., 2006; Diskin et al., 2012).

Both the N-terminal and C-terminal CRDs of galectin-8, as well as the hinge region connecting them, are required for optimal cell adhesion (Levy et al., 2006). It has further been shown that diverse cells interact with galectin-8 via subset of glycosylated integrins (Cárcamo et al., 2006; Diskin et al., 2009; Elola et al., 2014; Hadari et al., 2000) or other receptor classes, e.g. ALCAM (also known as CD166) (Fernández et al., 2016; Ferragut et al., 2019; Troncoso et al., 2014), podoplanin (Bieniasz-Krzywiec et al., 2019; Cueni and Detmar, 2009; Troncoso et al., 2014) and urokinase plasminogen-activated receptor (uPAR) (Vinik et al., 2015).

Information about cell spreading behavior on galectin-8 is, however, still limited. Specifically, the cytoskeletal reorganization, formation of lamellipodial and filopodial protrusions, and adhesion mechanics upon cell plating on galectin-8 have, as yet, been insufficiently studied. Even less is known about cell reactions to composite substrates that contain galectin-8 together with other matrix proteins, such as fibronectin, and some data suggest that mixtures of these proteins enhance the spreading of metastatic cell (Reticker-Flynn et al., 2012).

In this study, we compared, in detail, the process of cell adhesion and spreading on galectin-8 and on fibronectin. We found major differences in self-organization of the actomyosin cytoskeleton, formation of lamellipodial and filopodial protrusions, and assembly of ECM adhesions upon cell interaction with these two matrix proteins. These differences are attributed to the much stronger adhesion forces on galectin-8, and to different effects on small Rho family GTPases and their downstream targets. Furthermore, composite substrates that contain both proteins at different ratios, revealed reciprocal effects of galectin-8 and fibronectin upon stimulation of cell spreading and focal adhesion formation, respectively; yet, we noticed a strong synergy between the two in the formation of adhesive filopodial protrusions.

## RESULTS

### Different dynamics of filopodia and lamellipodia extensions of cells grown on fibronectin and galectin-8

**Fig. 1.**
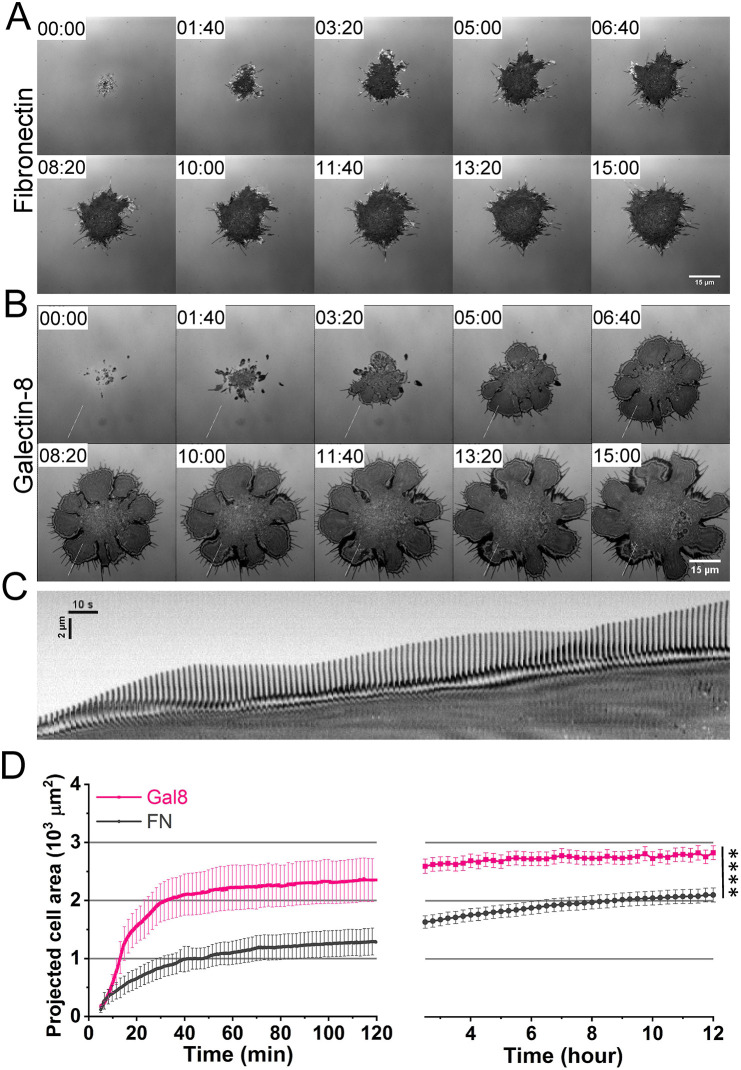
**Spreading of HeLa-JW cells on fibronectin and galectin-8-coated substrates.** (A,B) Time course of cell spreading in serum-free medium on substrates coated with fibronectin (A) or galectin-8 (B), imaged using interference reflection microscopy (IRM). Scale bars: 15 µm. Time is indicated in minutes (see also Movies 1–3). Notice that, due to faster extension of lamellar petals, the projected cell area on galectin-8 coats dramatically exceeds that on fibronectin coats. (C) Kymograph showing the time course of filopodia and lamellipodia protrusions within an area that is 1 µm wide and 16 µm long, crossing the cell periphery perpendicularly to the lamellipodia front, as indicated in B. Notice the waves of filopodia and lamellipodia extensions. (D) Quantification of projected cell area on fibronectin and galectin-8 at different time points after cell plating. Error bars indicate the standard errors of mean (±s.e.m.). Data of six cells under each condition were used in the spreading quantification at time points before 2 h (left), and 20 cells under each condition were measured to quantify cell spreading after 2.5 h (right). These results are based on four independent experiments. Two-sample two-tailed *t*-test was performed on the projected cell area after 2 h. ****P<0.005.

HeLa cell spreading manifests itself primarily in the continuous extension of two types of membrane protrusion, filopodia and lamellipodia. We used time-lapse interference reflection microscopy (IRM) (Barr and Bunnell, 2009) and differential interference contrast (DIC) microscopy to visualize these processes (Fig. 1A,B; Movies 1–3). On fibronectin, the spreading process was initiated by the formation of filopodia (Fig. 1A; Movie 1). The filopodia were succeeded by blebs and irregular lamellipodial protrusions that, when extended, paused or sometimes retracted (Movie 1). Usually, the lamellipodial spreading comprised short periods of rapid protrusion, alternating with prolonged pause periods (Fig. S1A), such that the net protrusion speed at the edge of the cell was slower than that of individual lamellipodia (Fig. 1D; Movie 1).

On galectin-8, cell spreading was also initiated by the formation of filopodia (Fig. 1B,C; Movies 2 and 3), which was rapidly succeeded by the formation of lamellipodia. The speed of lamellipodial extension was somewhat slower on galectin-8 compared with that on fibronectin (Fig. S1A). However, the extension of lamellipodia proceeded continuously without pauses (Fig. 1B; Fig. S1A; Movies 2 and 3). As a result, the net increase of projected cell area on galectin-8 happened considerably faster compared to that on fibronectin (Fig. 1D). When the velocity of lamellipodia growth decreased, filopodial growth increased in speed, and new filopodia extending from the lamellipodial edge often formed (Fig. 1C). Similarly, cessation of filopodial growth was accompanied by an increase in the rate of lamellipodial extension. Thus, on galectin-8, spreading was not intermittently stalled, and proceeded via alternating waves of lamellipodial and filopodial extensions (Fig. 1C; Movies 2 and 3). Another peculiar feature of cell spreading on galectin-8 was the petaloid cell contour. Extension of lamellipodia and filopodia occurred independently in 3–8 ‘petals’ (segments) of the cell periphery (Fig. 1B; Movies 2 and 3). This petaloid appearance was characteristic of the early stages of spreading on galectin-8. Later, neighboring petals would fuse and spreading became more isotropic.

### The actomyosin cytoskeleton organization differs in cells spreading on fibronectin and galectin-8

**Fig. 2.**
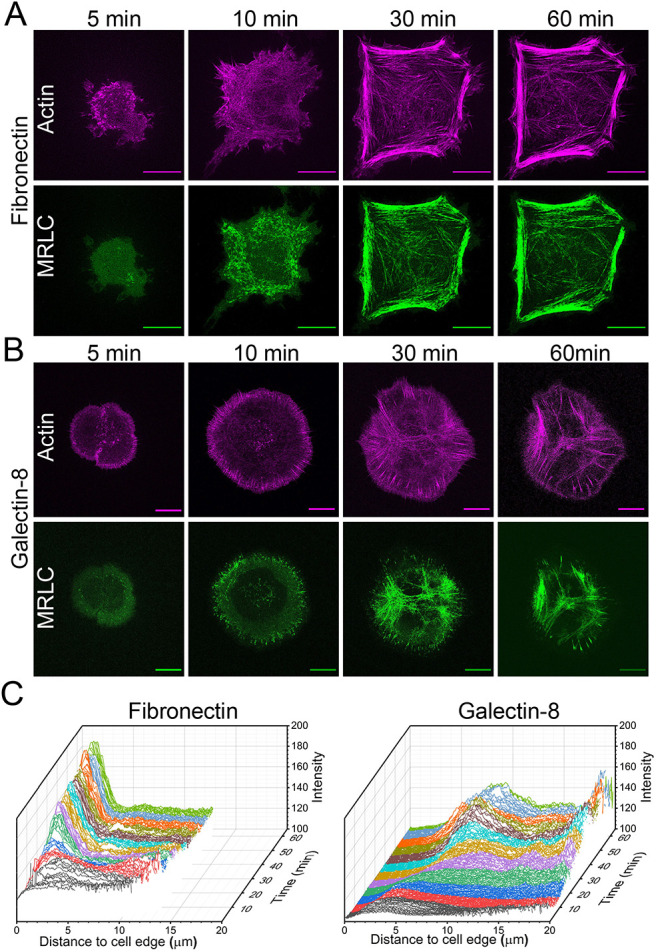
**Actin and myosin II dynamics during cell spreading.** (A,B) Time course of spreading of cells transfected with tdTomato–F-tractin (actin, pink) and GFP myosin II regulatory light chain (MRLC, green) and grown on fibronectin (A) or galectin-8 (B) (see also Movies 4 and 5). Scale bars: 10 µm. Ten minutes after plating, numerous non-organized myosin filaments appeared on both substrates. Notice the formation of prominent actin- and MRLC-containing fibers at the periphery of the cells spreading on fibronectin (A), and actomyosin arrays in cells spreading on galectin-8 (B). (C) Total intensity of GFP-MRLC fluorescence as a function of the distance from the cell edge, in cells plated on fibronectin (left) or on galectin-8 (right). Intensity profiles that correspond to different time intervals are colored arbitrarily to assist visual assessment.

In agreement with previous studies (Gauthier et al., 2012; Wolfenson et al., 2014), we confirmed that the main cytoplasmic extensions that formed during spreading on fibronectin were actin-rich lamellipodia and filopodia. Formation of these structures was followed by assembly of the actomyosin stress fiber system (Fig. 2A; Movie 4). The myosin II filaments visualized by cell transfection with GFP-myosin light chain appeared at the cell periphery and moved centripetally (Hu et al., 2017). Later, the myosin filaments concentrated in large stress fiber-like actin bundles delineating the edges of polygonal cells (Fig. 2A; Movie 4).

On galectin-8-coated substrates, cells displayed actin- and fascin-positive adherent filopodia around the cell periphery, which were denser, although not necessarily longer, than those on fibronectin-coated substrates (Fig. 2B; Fig. S1B). Compared with those on fibronectin, lamellipodia on galectin-8 were substantially more enriched with F-actin and occupied the entire cell periphery of circular or petaloid cells (Fig. 2). Tiny actin bundles were shown to grow centripetally from the cell edges (Fig. 2B), and some of these bundles appeared to constitute the core actin bundles of filopodia. The fine cytoskeletal organization within lamellipodia and filopodia, visualized by using cryo-electron tomography, clearly showed the extended ‘filopodial roots’ consisting of parallel actin filaments tailed into the lamellipodium (Fig. S1C). Although the peripheral parts of filopodial bundles were closely associated with the ventral membrane (characteristic distance from the substrate of 40–80 nm), the intra-lamellipodial parts of these bundles were usually located at the dorsal aspect of the lamellipodial actin meshwork (Fig. S1C).

On galectin-8-coated substrates, the myosin II filaments first appeared at the cell periphery, at a rate similar to those seen on the fibronectin-coated substrate (Movie 5); however, circumferential actomyosin bundles lying parallel to the cell edges were not detected. Instead, a star-like system of myosin II-enriched actin structures appeared in the central region of the cells (Fig. 2B; Movie 5). Quantification of the density of GFP-tagged myosin II regulatory light chain (MRLC)-containing filaments confirmed that, on fibronectin-coated substrates, the myosin II filaments were located at the cell periphery, whereas on galectin-8-coated substrates they concentrated at the cell center (Fig. 2C).

### Cells on galectin-8 do not form mature focal adhesions

**Fig. 3.**
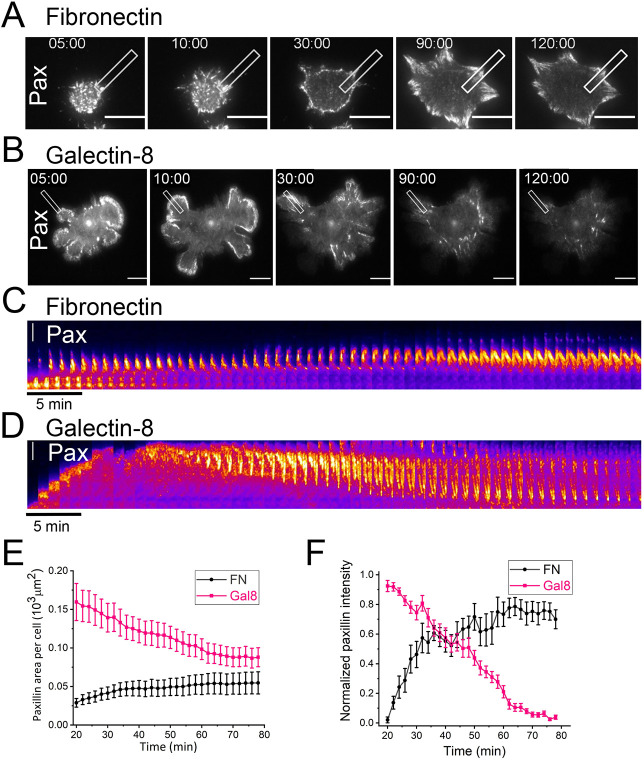
**Paxillin dynamics during cell spreading on fibronectin** **or** **galectin-8.** Sequences of images of cells, stably expressing YFP-paxillin, while spreading on fibronectin (A) or galectin-8 (B) (see Movies 6 and 7). Scale bars: 15 µm. Notice the formation and growth of focal adhesions in A and the formation of centripetal movement and disappearance of paxillin clusters in B. (C,D) High magnification kymographs showing paxillin dynamics in the rectangular areas (3×19 µm), drawn across the periphery of the cell, perpendicular to the cell edge in cells plated on fibronectin (C) or on galectin-8 (D). The *x*-axis represents time, the frames are taken every minute. The *y*-axis shows the position of the paxillin clusters. Scale bars: 5 µm. (E,F) Dynamics of the total area of paxillin clusters (E) and normalized total intensity of YFP-paxillin fluorescence (F) per cell, for cells plated on fibronectin (black) or galectin-8 (pink). Error bar shows the standard error of mean (±s.e.m.). Number of assessed cells: *n*=20, with results based on three independent experiments.

To investigate focal adhesion dynamics during the initial stages of cell spreading, we utilized HeLa-JW cells stably transfected with YFP tagged to paxilin (YFP-paxillin) and transiently transfected with mCherry-Lifeact to visualize F-actin, and imaged the cells by using TIRF microscopy. On fibronectin, the cells formed focal adhesions that underwent maturation (Fig. 3A,C; Movie 6), as described in many previous studies (Gardel et al., 2010; Geiger et al., 2009; Wolfenson et al., 2009). Maturation of focal adhesion is manifested by an increase in its area, as well as an increase in the fluorescence intensity of paxillin (Fig. 3A,C,E,F; Movie 6).

On galectin-8, evolution of paxillin-enriched complexes proceeded in an entirely different manner. At the early stage of spreading, relatively large clusters of paxillin were located at the periphery of actin-rich lamellipodial protrusions (Fig. 3B; Movie 7; Fig. S1D). When the rate of lamellipodial extensions slowed, the paxillin clusters disintegrated into smaller patches that moved centripetally and formed radially oriented threads associated with thin actin fibers (Fig. 3D; Fig. S1D). During the retrograde movement, paxillin clusters decreased in size and brightness, and gradually disappeared (Fig. 3B,D; Fig. S1D). Two hours after cell plating, most of the paxillin structures had vanished (Fig. 3B). Compared with cells grown on fibronectin, quantification of the areas and fluorescence intensity of paxillin clusters formed by cells grown on galectin-8 clearly illustrate the differences in the dynamics of paxillin-containing structures on these two substrates (Fig. 3E,F).

**Fig. 4.**
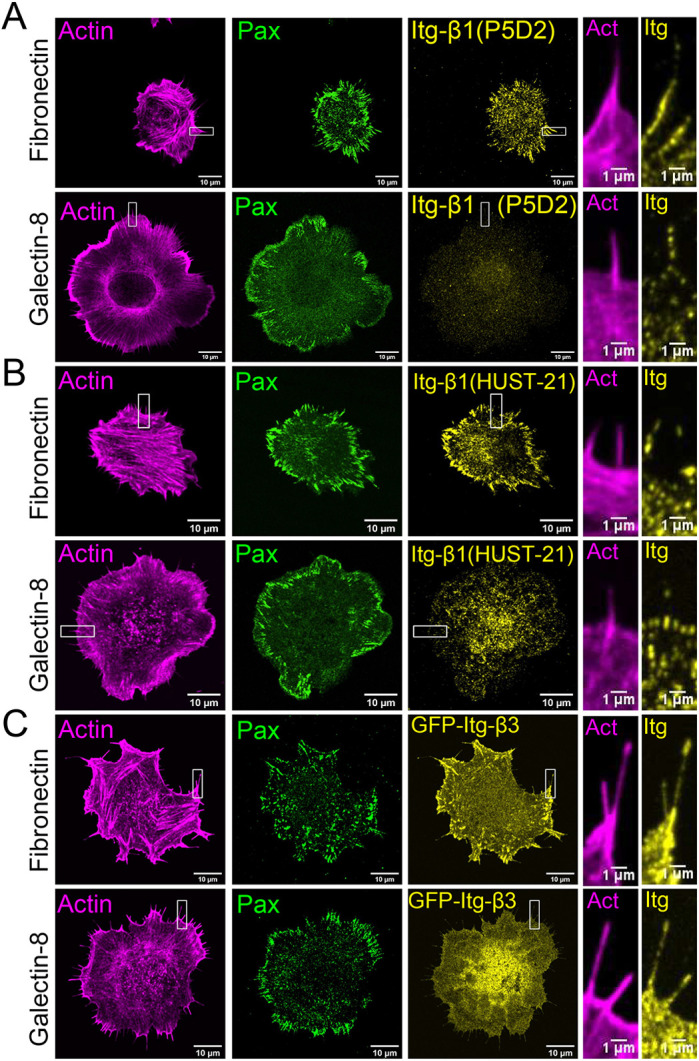
**Integrin localization in cells spreading on fibronectin** **or** **galectin-8.** (A,B) HeLa-JW cells, stably expressing YFP-paxillin, stained using integrin β1 antibodies P5D2-s (A) and HUST21 (B). (C) Visualization of integrin in B16 melanoma cells stably expressing GFP-integrin-β3. Actin was labeled by Tetramethylrhodamine (TRITC)-conjugated phalloidin (left column). Filopodia labeling within the boxed areas of the actin and integrin images are shown at higher magnification in the two columns on the right. Notice that integrin β1 antibody labeling colocalizes with that of YFP-paxillin in cells grown on fibronectin but not in cells grown on galectin-8 (A,B). Similarly, GFP-integrin-β3 in B16 melanoma cells colocalizes with paxillin antibody staining in cells grown on fibronectin but not in cells grown on galectin-8 (C). Overall, filopodia often contain integrins β1 or β3 in cells grown on both fibronectin- or galectin-8-coated substrates.

Paxillin-containing clusters formed by cells during spreading on galectin-8 contained several other adhesion-plaque proteins, i.e. talin, zyxin and VASP (Fig. S2; Movie 8). These clusters were also enriched in proteins phosphorylated at tyrosine residues, which is similar to focal adhesions in cells growing on fibronectin (Fig. S2). Strikingly though, unlike focal adhesion, paxillin-containing clusters were not enriched with integrin β1 or activated integrin β1, as shown by staining with corresponding antibodies (Fig. 4A,B). To further check whether the paxillin clusters formed on galectin-8 substrates contain integrin β3, we used B16 melanoma cells stably expressing GFP-integrin-β3. These cells displayed a different spreading behavior on galectin-8 and fibronectin, comparable to that of Hela-JW cells; in particular, they formed considerably more filopodia on substrates coated with galectin-8 than on those coated with fibronectin (Fig. S3E). Twenty minutes following plating cells on galectin-8-coated substrate, cells formed paxillin clusters similar to those formed by Hela-JW cells (Fig. 4C; Fig. S3E). However, on fibronectin-coated substrates, B16 melanoma cells formed focal adhesions enriched with GFP-integrin-β3 (Fig. 4C), whereas paxillin-rich clusters formed on galectin-8 did not contain GFP-integrin-β3 (Fig. 4C), and were associated with short actin filaments, rather than with the ends of stress fibers. At the same time, it was possible to detect integrins β1 and β3 at the tips of some of the adherent filopodia formed by the cells when plated on galectin-8 (Fig. 4A–C).

In addition to B16 melanoma cells, we also examined the spreading of several other cell types on galectin-8-coated substrates. Cells tested included primary murine cardiac fibroblasts, human dermal fibroblasts (HDF), osteosarcoma (U2OS), fibrosarcomas (HT1080) and rat embryo fibroblasts (REF-52). An increase in the projected cell area on cells grown on galectin-8 was detected in primary murine cardiac fibroblast, U2OS and REF-52 cells (Fig. S3). Both B16 melanoma and HT1080 cells formed more filopodia on galectin-8-coated than on fibronectin-coated substrates. All cells examined demonstrated deficient formation and maturation of focal adhesions (Fig. S3). In particular, GFP-integrin-β3-expressing REF-52 cells formed numerous integrin-β3-positive focal adhesions on fibronectin-coated substrates but did not form any on galectin-8-coated substrates.

The two CRDs of galectin-8 are not equal (Cagnoni et al., 2020). In agreement with Levy et al. (2006), our experiments with galectin-8 CRD plus hinge deletion mutants mutants revealed that both the N- and C-terminal CRDs, as well as the extended hinge region between these domains, are necessary to induce the full galectin-8-spreading phenotype that is characterized by extended formation of lamellipodia. Cells attached only very weakly to galectin-8 that lacks the N-terminal CRD (Gal8-C) (Fig. S4A). On galectin-8 that lacks the C-terminal CRD (Gal8-N) or the hinge region (Gal8-Δhinge), cells still attached but mainly formed filopodia and/or retracting fibers, they did not form lamellipodia (Fig. S4A). Thus, only full-length galectin-8 can trigger the complete cellular response during cell attachment and spreading.

In addition to the differences in the assembly of the actomyosin cytoskeleton and focal adhesions on fibronectin and galectin-8-coated substrates, we revealed that the strength of the adhesion that developed immediately after cells attached to the galectin-8-coated substrate was significantly higher than that to the fibronectin substrate.

**Fig. 5.**
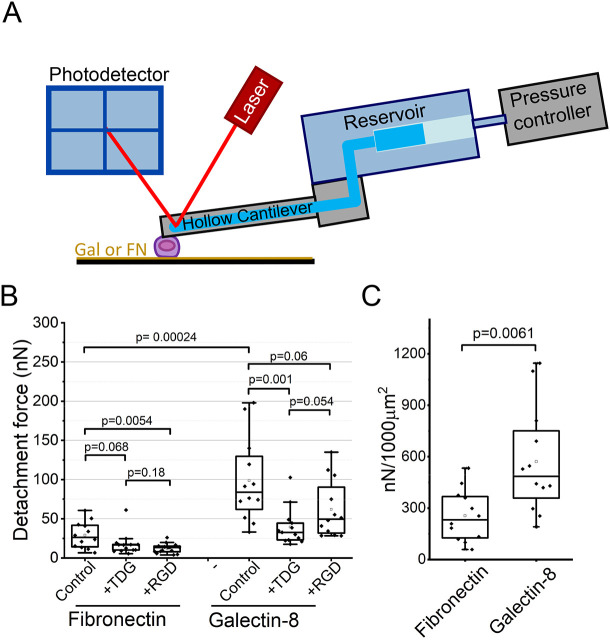
**Cell-substrate adhesion forces on fibronectin** **or** **galectin-8 during early cell spreading.** (A) Schematic depicting the set-up of the microfluidic device used to measure adhesion forces. A pulling force is applied to the cell by a vacuum system integrated with the AFM cantilever. Adhesion force is defined as the minimal force sufficient to detach the cells from the substrate. (B) Measurement of cell-substrate adhesion forces in HeLa-JW cells 5 min after plating them on either fibronectin or galectin-8 in serum-free medium (control), or in the serum-free medium containing 20 mM TDG (homologue of β-galactoside) or 10 µg/ml RGD peptide (CGGRGD). (C) Adhesion forces on fibronectin and galectin-8 substrates, normalized per projected cell area (µm^2^). For each condition, 12–13 cells were measured. *P* values were calculated using two tailed Mann–Whitney test. Notice that adhesion forces per cell (B) and per unit of cell area (C) were significantly higher on galectin-8 than on fibronectin. On the fibronectin substrates, excess of either RGD or TDG decreased the adhesion force, whereas on galectin-8 substrate only treatment with TDG, but not with RGD, reduced adhesion. Data are presented as box and whisker plots, showing median values, upper and lower quartiles, maximum and minimum, and outliers (values that are 1.5 times larger than the upper or 1.5 times smaller than the lower quartiles).

We assessed the forces required to detach cells from each substrate by using FluidFM technology in which the AFM cantilever was supplied with a microfluidic channel that permitted immobilization of the cell at the cantilever by applying negative pressure (Fig. 5A). The cell immobilized on the cantilever was allowed to contact the substrate, and the moment of initial contact was detected by cantilever deflection. Five minutes following the initial contact, the cells were detached from the substrate by uniaxial retraction of the cantilever. During this process, the deflection of the cantilever proportional to the applied force was recorded, and the maximum detachment force (MDF) was extracted as a representative parameter characterizing cell adhesion (Sancho et al., 2017). We found that the forces required to detach cells from the galectin-8-coated substrate were dramatically higher than those required to detach cells from the fibronectin-coated substrate (Fig. 5B). Furthermore, 5 min after the initial contact, the average cell spreading area on galectin-8 was larger than that seen on the fibronectin-coated substrate (172.9 μm^2^ vs 113.5 μm^2^). To determine the adhesion force per unit of cell substrate interface, we normalized MDF values to the mean cell-projected area on the corresponding substrates (Fig. 5C). The adhesion force per unit of cell area was still much higher for cells spreading on galectin-8 than for cells spreading on fibronectin (Fig. 5C).

As predicted, addition of an Arg-Gly-Asp (RGD)-containing peptide, which competes with integrin binding to fibronectin, considerably reduced the force required for cell detachment from fibronectin-coated substrate after 5 min of contact, but only weakly affected the force necessary to detach cells from galectin-8-coated substrates (Fig. 5B). Excess of RGD reduced also the projected cell area and focal adhesion formation on fibronectin-coated substrates after 30 min of spreading, but did not affect the projected cell area and paxillin clusters on galectin-8 (Fig. S4B,C), which is consistent with the aforementioned data, showing that integrin β1 and β3 did not incorporate into the matrix adhesions on galectin-8 (Fig. 4).

Addition of β-galactoside homologue TDG, which blocks the CRD of lectins (Kaufman and Lawless, 1980) including galectin-8 (Delaine et al., 2008) during the initial 5 min of attachment, strongly reduced the cell MDF on galectin-8-coated substrates, but also reduced such forces on fibronectin-coated substrates (Fig. 5B). However, after 30 min of spreading, TDG dramatically reduced the projected area of cells spreading on galectin-8, but did not reduce the projected cell area and paxillin-positive focal adhesions on fibronectin (Fig. S4D,E).

### Effects of experimental manipulations with small Rho GTPases on cells spreading on galectin-8 and fibronectin-coated substrates

Small GTPases of Rho family are the master regulators of the actin cytoskeleton. Their activation downstream of interactions of cells with the extracellular matrix is thought to determine the processes of cell adhesion, spreading and polarization upon the cell contact with the matrix. To elucidate the mechanism of cell response to fibronectin and galectin-8-coated substrates, we investigated the functions of three main Rho GTPases, RhoA, Rac1 and Cdc42 during cell spreading on these two substrates.

**Fig. 6.**
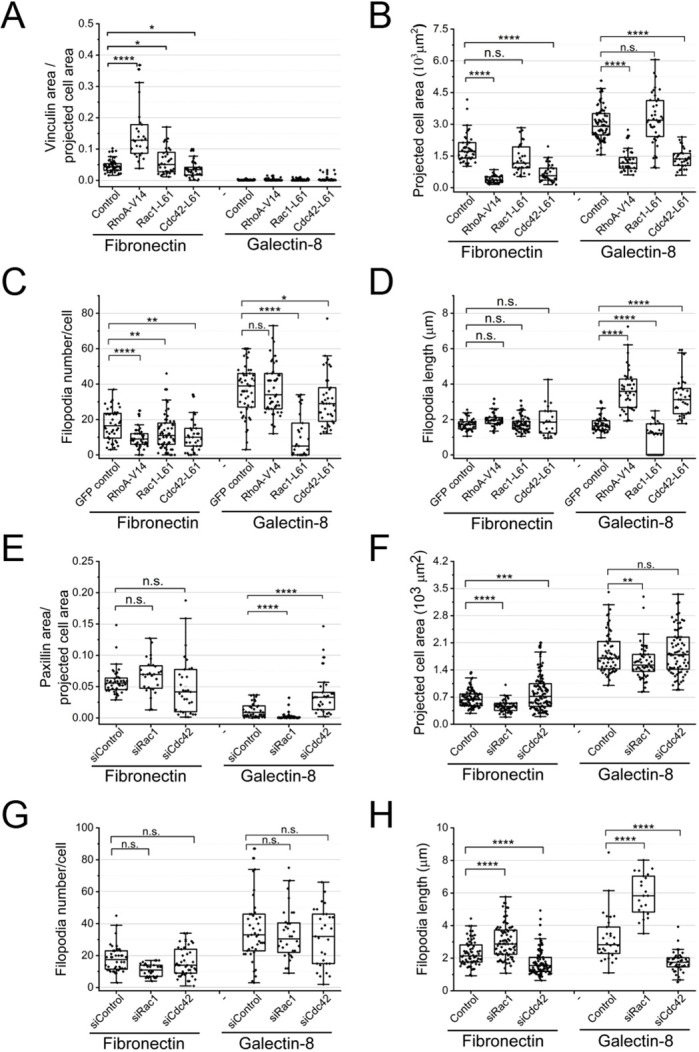
**Effects of small Rho GTPases** **regarding** **cell spreading on fibronectin-coated** **or** **galectin-8-coated substrates.** (A–D) Focal adhesions, projected cell area and filopodia in control HeLa-JW cells, and in cells transfected with constitutively active small Rho GTPases, GFP-RhoA-V14, GFP-Rac1-L61 or GFP-Cdc42-L61, assessed 2 h after plating on fibronectin- or galectin-8-coated substrates. (E–H) Similar parameters assessed in control, Rac1-knockdown and Cdc42-knockdown cells 30 min after plating. Focal adhesion area as a fraction of total project cell area (A,E), projected cell area (B,F), filopodia number per cell (C,G), average length of filopodia per cell (D,H). Cells, fixed and stained with TRITC-phalloidin and antibodies for vinculin (A–D) or paxillin (E–H), were used for morphometric measurements. Morphometric measurements and presentation of results were performed as described in the legend to Fig. 5. In all graphs, each dot corresponds to an individual cell. *n*≥40 cells were assessed under each experimental condition, and the experiments were repeated three times. *P* values were calculated using two-sample two-tailed *t*-tests. *P<0.05, **P<0.01, ***P<0.005, ****P<0.001; n.s., not significant.

We assessed how activation or depletion of small Rho GTPases, as well as of their downstream targets, affects cell spreading on both substrates. The quantitative parameters chosen to characterize cell spreading were: (1) the cell-projected area, reflecting bulk protrusion activity of lamellipodia; (2) the size of paxillin- and/or vinculin-positive adhesion structures as a fraction of cell area and; (3) the number and average length of adherent cellular filopodia (Fig. 6).

Activation of RhoA by either expressing the constitutively active RhoA mutant RhoA-V14 or by addition of its pharmacological activator CN03 (Cytoskeleton, Inc.) (Flatau et al., 1997; Schmidt et al., 1997) significantly promoted formation of vinculin- or paxillin-positive focal adhesions and linear actin stress fibers on fibronectin, but did not seem to protect vinculin and/or paxillin clusters from disappearance on galectin-8 (Fig. 6A; Fig. S5C,D,G). The projected area of cells on fibronectin decreased upon RhoA activation, whereas, on galectin-8 – despite RhoA activation – the projected cell area always remained larger than that on fibronectin (Fig. 6B; Fig. S5D,G). Constitutively active RhoA promoted formation of filopodia on the galectin-8-coated substrate (Fig. 6C,D; Fig. S5D; Movie 9). These stimulated filopodia were much longer than those seen in control cells (Fig. 6D) and often displayed triangular actin-enriched ‘pedestals’ at their bases (Fig. S5D′).

Expression of constitutively active Rac1 (mutant Rac1-L61) resulted in a circular cell shape, promoting lamellipodia formation on both substrates (Fig. S5E; Movie 10). Although it led to the formation of radial and circumferential actin bundles associated with enlarged focal adhesions on fibronectin, expression of Rac1-L61 did not rescue the formation of focal adhesion on galectin-8 (Fig. 6A; Fig. S5E). The difference in the spreading area between cells on fibronectin and galectin-8 did not decrease upon Rac1 activation (Fig. 6B). At the same time, constitutively active Rac1 strongly suppressed filopodia formation on galectin-8 (Fig. 6C,D; Fig. S5D,D′).

Expression of the constitutively active Cdc42 mutant Cdc42-L61 decreased lamellipodia formation and spreading area on both substrates (Fig. 6B; Fig. S5F), but it did not rescue the formation of focal adhesions and stress fibers on the galectin-8-coated substrate (Fig. 6A; Fig. S5F). Similarly to active RhoA, constitutively active Cdc42-L61 augmented filopodia length on galectin-8, promoting formation of actin-enriched pedestals (Fig. 6C,D; Fig. S5F,F′).

We then studied the effects of Rac1 and Cdc42 knockdown regarding cell spreading on fibronectin and galectin-8-coated substrates. On fibronectin, knockdown of either Rac1 or Cdc42 did not significantly affect the fraction of cell area occupied by paxillin-positive adhesions (Fig. 6E). In Rac1-knockdown cells grown on galectin-8, paxillin clusters that had formed at the initial stages of spreading were reduced, whereas in Cdc42-knockdown cells grown on galectin-8, these clusters became larger compared with those in control cells (Fig. 6E; Fig. S6E-G). In addition, when spreading on galectin-8, Cdc42-knockdown cells formed paxillin clusters that did not disassemble within 4 h following plating (Fig. S6E′-G′), unlike control cells (Fig. S6A′). Knockdown of Rac1 decreased the cell spreading area on fibronectin and galectin-8 (Fig. 6F; Fig. S6B-D), whereas knockdown of Cdc42 slightly increased the cell spreading area on fibronectin but did not change on galectin-8 (Fig. 6F; Fig. S6). Clear differences were observed between the projected cell area of control cells, and Rac1- and Cdc42-knockdown cells grown on fibronectin or galectin-8 (Fig. 6F). Neither Rac1- nor Cdc42-knockdown did significantly change the number of filopodia (Fig. 6G; Fig. S6). However, Rac1-knockdown did increase filopodia length on both substrates, and knockdown of Cdc42 reduced filopodia length on either substrate (Fig. 6H; Fig. S6).

**Fig. 7.**
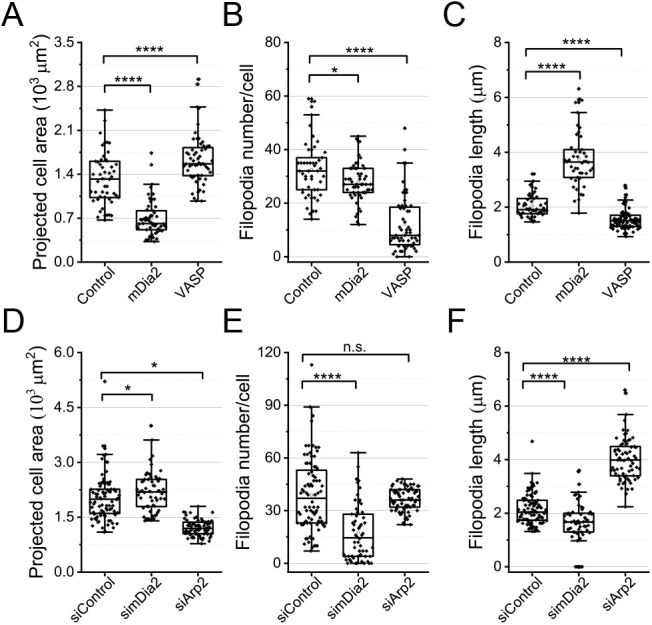
**Effects of actin polymerization regulators on cells** **spreading on galectin-8.** (A–C) Control cells and cells overexpressing GFP-mDia2 or GFP-VASP. (D–F) Control cells and cells expressing siRNAs for mDia2 or Arp2. Cells were plated on galectin-8-coated substrates in serum-free medium and fixed 20 min later. Cells were stained with TRITC-phalloidin. Morphometric measurements and presentation of results were performed as described in the legend to Figs 5 and 6. Each dot corresponds to an individual cell; *n*≥40 cells were assessed for each experimental condition, and the experiments were repeated three times. *P* values were calculated using two tailed *t*-tests. *P<0.05, ****P<0.001; n.s., not significant.

Among downstream effectors of small GTPases, we investigated the effect activation or depletion of actin polymerization regulators, the Arp2/3 complex and the formin Diaph3 (hereafter referred to as mDia2), has regarding early cell spreading on galectin-8. In addition, we assessed the effects of pharmacological inhibition of the RhoA target, Rho kinase (ROCK). Expression of constitutively active construct of mDia2 decreased projected cell area (Fig. 7A; Fig. S7A,B), did not increase filopodia number, but increased filopodia length (Fig. 7B,C). Consistently, knockdown of mDia2 increased the projected cell area and suppressed formation of filopodia (Fig. 7D,E; Fig. S7E). These effects are in contrast to those caused by depletion of the Arp2/3 complex when Arp2 was knocked down, i.e. Arp2/3 depletion significantly decreased the projected cell area, presumably by inhibition of lamellipodia (Fig. 7D; Fig. S7E) and enhanced elongation of filopodia (Fig. 7F; Fig. S7E). Finally, inhibition of ROCK in response to its specific inhibitor Y27632 increased the projected cell area on both fibronectin and galectin-8. However, the projected area of cells grown on galectin-8 remained much larger than that seen on fibronectin-grown cells (Fig. S7F, left). In addition, Y27632 treatment reduced the area of paxillin clusters on both substrates (Fig. S7F, middle), and the number of filopodia on galectin-8 (Fig. S7F, right).

### Combined effects of fibronectin and galectin-8 on cell spreading, focal adhesion and filopodia formation

**Fig. 8.**
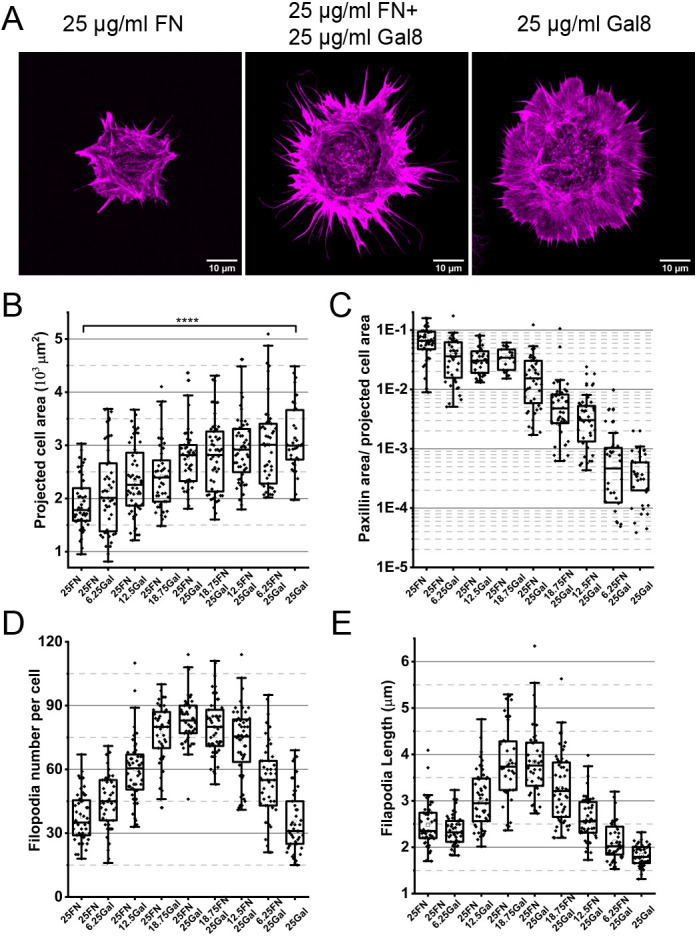
**Cell spreading on composite substrates comprising fibronectin and galectin-8 at different ratios.** (A) F-actin visualized by TRITC-phalloidin staining in cells fixed 20 min after plating on substrates coated with 25 µg/ml fibronectin, a combination of 25 µg/ml fibronectin and 25 µg/ml galectin-8 or 25 µg/ml galectin-8. (B-E) Quantification of projected cell area (B), total paxillin adhesion area (C), filopodia number per cell (D) and average filopodia length per cell (E) for cells spreading on the composite substrates. The numbers indicate the concentration (µg/ml) of each protein in the incubation buffer used for coating the substrates. Note that the adsorption of fibronectin at low concentrations (≤15 µg/ml) is somewhat reduced in the presence of high galectin-8 concentrations (see Fig. S8E). Each dot corresponds to an individual cell; *n*≥40 cells were assessed under each experimental condition. These results are based on three independent experiments. Results are presented as a box and whisker plot, as in Figs 5 and 6. *P* values were calculated using two-sample two-tailed *t*-tests.

*In vivo*, cells only rarely, if at all, encounter an extracellular matrix consisting of one type of protein. As a rule, cell behavior is determined by a complex mixture of extracellular ligands. Here, we studied a simplified situation, analyzing the cellular response to a mixture of two types of matrix protein, i.e. fibronectin and galectin-8, when employed at different ratios. A time point of 4 h was chosen to assess the cells at maximal spreading. At this time point, the average spreading area on fibronectin had the maximal value of ∼1700 µm^2^. The substrate coated with a mixture of fibronectin at a maximal concentration (25 µg/ml), and an increasing concentration of galectin-8 resulted in a gradual increase in cell spreading area up to ∼2800 µm^2^, seen at the maximal concentration of galectin-8 (25 µg/ml). Remarkably, a gradual decrease in the concentration of fibronectin together with the maximal concentration of galectin-8 led to an additional small, although significant, increase of the projected cell area (Fig. 8B; Fig. S8A). Thus, galectin-8 strongly regulates cell spreading in a positive manner, even when mixed with the maximal concentration of fibronectin. Fibronectin, however, exerts a slight negative effect on cell spreading at the maximal concentration of galectin-8.

The size of paxillin-containing complexes also changed in response to different ratios of fibronectin and galectin-8. Focal adhesions developed to the maximum extent at the highest concentration of fibronectin, and only slightly decreased when galectin-8 concentrations were increased (Fig. 8C; Fig. S8A). At the maximal concentration of galectin-8, a decrease in fibronectin concentration led to a decrease in the area of focal adhesions. However, the focal adhesions still existed even with the minimum fibronectin added. Indeed, staining using an antibody against human integrin β1 (P5D2) revealed that activated integrin β1 colocalized with paxillin in these structures (Fig. S8B). The focal adhesions on pure galectin-8 disappeared at this time point (4 h after seeding) (Fig. S8A). Thus, galectin-8 does neither support the formation of focal adhesions nor does it inhibit formation of focal adhesions in the presence of fibronectin. Even low concentrations of fibronectin can promote the formation of some focal adhesions in the presence of the maximal concentration of galectin-8.

Unlike the projected cell area that mainly responded to changes in the concentration of galectin-8, and unlike focal adhesions that mainly responded to changes in the concentration of fibronectin, formation of filopodia was sensitive to the concentrations of both ligands. On the substrate consisting solely of fibronectin, the number of filopodia per cell was relatively low (37 per cell, on average). Addition of increasing concentrations of galectin-8 prompted a marked increase in the numbers of protruding filopodia (>80 per cell) (Movie 11). At 4 h after plating on substrates covered by fibronectin and galectin-8 at several different ratios, the cells demonstrated numerous filopodia, some extending and others retracting (Fig. 8D,E; Movie 11). Under conditions of the highest galectin-8 concentration (25 µg/ml), the gradual decrease in fibronectin concentration (from 25 to 6.25 µg/ml) also led to a dramatic decrease in filopodia number (Fig. 8D,E). In addition, filopodia were synergistically enhanced on substrates coated by combinations of galectin-8 and fibronectin 30 min after spreading (Fig. 8A; Fig. S8C; Movie 12). Altogether, the dependence of filopodia number on the concentration of these two ligands demonstrates their synergistic effect, as the maximal number of filopodia was reached at the combined maximal concentrations of fibronectin and galectin-8. By contrast, decrease of either fibronectin or galectin-8 strongly decreased the number of filopodia. Of note, at optimal concentrations of fibronectin and galectin-8 (i.e. 25 µg/ml and 25 µg/ml, respectively), thus maximizing the number of filopodia, the mean filopodia length was also significantly increased compared with that at non-optimal concentrations (lower than 25 μg/ml for one or both of the two proteins) (Fig. 8E).

## DISCUSSION

In this study, we demonstrated that cell spreading on substrates covered by fibronectin and galectin-8 is strikingly different. The elementary processes of cell spreading include the formation of cell extensions, lamellipodia and filopodia, and adhesion of these extensions to the substrates. These processes are brought about by reorganizations of the actomyosin cytoskeleton. Lamellipodial and filopodial extensions are filled with an Arp2/3-nucleated branched actin network and formin-nucleated actin bundles, respectively (Blanchoin et al., 2014; Campellone and Welch, 2010; Chhabra and Higgs, 2007; Le Clainche and Carlier, 2008). Focal adhesion plaques are built of actin filaments and associated with actin bundles that transmit actomyosin-generated forces (Medalia and Geiger, 2010; Xia and Kanchanawong, 2017). The processes of cell spreading on a fibronectin-coated rigid planar substrate have been well studied. The projected cell area increases due to formation of lamellipodia, which triggers formation and maturation of focal adhesions associated with the systems of actomyosin bundles (stress fibers) (Small et al., 1998; Wolfenson et al., 2014). The subsequent reorganization of the stress fibers determines the rate of elongation and the polarization of the initially radial symmetric cells (Prager-Khoutorsky et al., 2011). For cells grown on galectin-8, all stages of this process differ. Formation of lamellipodia proceeds more persistently, without any episodes of pausing and retraction typically seen in cells spreading on fibronectin. Several lamellipodia are usually formed simultaneously at three to eight segments of the cell periphery, resulting in the development of a characteristic petaloid cell contour. Remarkably, cellular adhesion to a substrate coated with galectin-8 is much stronger than to one coated with fibronectin, as demonstrated in our measurement of forces per unit of projected cell area required to detach cells from the substrates. Five minutes after initial contact with the substrate, such forces are two-fold higher on the galectin-8- than on fibronectin-coated substrate.

Rapidly extending and strongly adherent lamellipodia on galectin-8 are, however, unable to form focal adhesions. When growing on fibronectin, cells form numerous dot-like nascent adhesions that, eventually, mature into the typical elongated focal adhesions associated with stress fibers (Burridge, 2017; Geiger et al., 2009). On galectin-8, however, cells initially form large paxillin-, talin- and vinculin-enriched clusters at the cell periphery, which then move centripetally and, eventually, disappeared. We examined the distribution of the prominent adhesion receptors integrin β1 and integrin β3, and found no evidence of integrin-β1 (activated or not) in the paxillin clusters. Similarly, stably expressed GFP-integrin-β3 does not colocalize with these structures. In contrast to cells spreading on galectin-8, both integrins β1 and β3 are detected in the focal adhesions of cells growing on fibronectin.

Accordingly, organization of the actomyosin fiber system proceeds differently on each of these two substrates. The peripheral actomyosin bundles, which exert traction forces on focal adhesions typical of cells spreading on fibronectin (Burridge and Guilluy, 2016; Kassianidou and Kumar, 2015; Tojkander et al., 2012), never form on galectin-8. Myosin II filaments still intensely assemble at the cell periphery during cell spreading but, instead of incorporating into circumferential actin bundles, they form several star-like contraction foci that are randomly located in the central part of the cell. Finally, cell spreading on galectin-8 involves the formation of numerous filopodia that extend from the edges of lamellipodia. Although cells spreading on fibronectin can also form filopodia – especially at the early stages of attachment – such filopodia often fail to adhere to the substrate and rapidly retract. Formation of numerous adherent filopodia at advanced stages of spreading is typically seen in cells that spread on galectin-8.

One, potentially important, mechanism underlying the differences in cells spreading seen on fibronectin and galectin-8 might depend on varying downstream signals from the adhesion receptors. Since the receptors involved in cell adhesion to galectin-8 are numerous (Bieniasz-Krzywiec et al., 2019; Cárcamo et al., 2006; Cueni and Detmar, 2009; Elola et al., 2014; Fernández et al., 2016; Ferragut et al., 2019; Hadari et al., 2000; Troncoso et al., 2014; Vinik et al., 2015) and because only some of them have been identified, it is difficult to analyze differences between these signaling pathways in full detail. The lack of focal adhesion and stress fibers on galectin-8 could, in part, be attributed to failed integrin activation and downstream integrin signaling. We focus on the downstream level of signaling; namely, activation of the Rho family of small G proteins and their main cytoskeletal targets.

It is well-established that, on fibronectin-coated substrates, spreading is initiated by activation of Rac1 and Cdc42 that, in turn, triggers Arp2/3-dependent branched actin polymerization (Devreotes and Horwitz, 2015; Price et al., 1998). The integrin-mediated activation of RhoA at later stages of cell spreading on rigid fibronectin-coated substrates promotes myosin-II-driven actomyosin contractility, which restricts further spreading and triggers maturation of focal adhesions (Burridge and Guilluy, 2016; Burridge et al., 2019; Ren et al., 1999). Apparently, attachment of cells to galectin-8 also triggers the activation of small Rho GTPases (Cárcamo et al., 2006; Diskin et al., 2012). To elucidate the effects of these GTPases on phenotypical features of spreading cells, we performed several experiments that involved activation and depletion of these GTPases. On galectin-8, the projected cell area can be reduced by overexpression of constitutively active RhoA or Cdc42. These effects could be explained by suppression of lamellipodia formation and/or increase of overall cell contractility by RhoA and Cdc42. Indeed, both RhoA and Cdc42 can activate myosin II light chain phosphorylation through their immediate targets ROCK and MRCK, respectively (Wilkinson et al., 2005; Zhao and Manser, 2015). Accordingly, we showed that inhibition of ROCK increases the projected cell area on galectin-8.

Constitutively active Cdc42 or RhoA mutants increased filopodia length on galectin-8, whereas knockdown of Cdc42 or inhibition of ROCK decreased it. At the same time, Rac1 has an antagonistic effect on filopodia formation in cells grown on galectin-8, since its constitutively active mutant strongly reduces filopodia number and length, whereas its depletion augments filopodia. Effects of small G-proteins on cell projected area and filopodia formation are consistent with the effects of their immediate targets, regulators of actin polymerization, the Arp2/3 complex and formins. Overexpression of the formin mDia2, a downstream target of RhoA and Cdc42 (Kühn and Geyer, 2014), activates formation of filopodia but decreases cell projected area. Knockdown of mDia2 decreased the number of filopodia and increased the projected cell area. Moreover, knockdown of Arp2, which suppresses Arp2/3–actin polymerization, decreases the projected cell area but, in agreement with previous studies promoted filopodia elongation by augmenting their length (Innocenti, 2018; Steffen et al., 2014; Swaney and Li, 2016).

Thus, the overall formation pattern of actin-rich extensions and cell spreading on galectin-8-coated substrates is likely to arise out of the interplay between two competing processes: the Arp2/3-driven assembly of the branched actin network, which results in formation and extension of lamellipodia, and the formin-driven assembly of filopodia actin cores. These two processes are antagonistic, since they compete for the same pool of monomeric actin. Similar to what is seen in other experimental systems (Innocenti, 2018; Steffen et al., 2014), Arp2/3-driven actin polymerization in the lamellipodia on galectin-8 is activated by Rac1. Our study suggests that formin-driven actin polymerization in filopodia on galectin-8 is activated not only by Cdc42, which is broadly accepted (Mellor, 2010), but also by the RhoA-ROCK signaling axis, perhaps via the activation of mDia2.

Deficiency in the formation of focal adhesions cannot be rescued by manipulations with Rho and Rac1. Interestingly, elimination of paxillin clusters in cells grown on galectin-8, can be prevented partially by depletion of Cdc42, which indicates participation of Cdc42 in suppressing the formation of focal adhesion on galectin-8.

Importantly, the differences in the projected cell area between galectin-8 and fibronectin cannot be fully explained by activity changes of small Rho GTPases and their targets. Even though it can be reduced upon activation of RhoA, Cdc42 and formins, as well as upon inhibition of Rac1 and the Arp2/3 complex, the projected cell area on galectin-8 still always exceeds the projected cell area on fibronectin. This suggests that other factors should also be taken into consideration; most probably these are the strength of physical adhesions detected in our study.

Cell spreading on mixtures of fibronectin and galectin-8 in varying proportions, revealed the ability of cells to combine the signals produced by contact with different components of the ECM, and to determine their different effects on cell spreading, focal adhesion formation and filopodia extension. Galectin-8 increased the projected area in a concentration-dependent fashion when fibronectin was present at its highest concentration. The extent and rate of cell spreading on a matrix comprising a mix of the two matrix proteins is dominated by galectin-8 and only moderately – negatively – regulated by fibronectin. Similarly, formation of focal adhesions on the mixed matrix is dominated by fibronectin, as it induces these structures in a concentration-dependent manner, even in the presence of the highest concentration of galectin-8. The latter only slightly reduced the area of focal adhesions in the presence of the highest concentration of fibronectin. Unlike the spreading and formation of focal adhesions, extension of filopodia on a mix of fibronectin–galectin-8 substrate is considerably increased compared with that on either substrate individually. Both filopodia number and length increase upon addition of even a small amount of one ligand when the other ligand is present at maximal concentration. Maximal formation of filopodia was observed with both ligands present at maximal concentration. This finding demonstrates the possible synergistic effects of different extracellular matrix components, and might have important roles in cell adhesion and migration *in vivo.* In particular, since formation of filopodia has been shown to correlate with cancer cell metastasis (Arjonen et al., 2011; Jacquemet et al., 2015), our results could explain why excessive production of galectin-8 augments the metastatic capacity of cancer cells (Gentilini et al., 2017; Reticker-Flynn et al., 2012; Shatz-Azoulay et al., 2020).

In summary, we demonstrated in this study that a galectin-8-coated substrate induces a cell adhesion response that differs from that induced through a fibronectin-coated substrate. Compared with cells grown on fibronectin, cells on galectin-8 spread more rapidly and persistently, and approach a larger projected area. This results from the increased efficiency of lamellipodia extension that, in turn, depends on activation of Arp2/3 as well as on stronger adhesion between cell receptors and the galectin-8 ligand. Cells plated on a galactin-8-coated substrate cannot, however, form integrin-containing mature focal adhesions and their associated system of actin stress fibers. RhoA-induced myosin II filaments in cells that spread on galectin-8 do not assemble into transverse arcs and ventral stress fibers typical for cells that spread on fibronectin. Rather, spreading on galectin-8 entails the robust formin-dependent formation of adherent filopodia triggered by Cdc42 and RhoA, and strongly opposed by Rac1-Arp2/3. Galectin-8-induced formation of filopodia is synergistically activated by fibronectin, such that filopodia number and length on the substrate coated with a mixture of both ligands dramatically exceed those on each type of ligand separately. Such synergistic effects may play an important role in the cellular response to composite matrices *in vivo*.

## MATERIALS AND METHODS

### Cell culture, DNA constructs and reagents

HeLa-JW cells were derived from the cervical carcinoma HeLa cell line in the laboratory of Jim Willams (Carnegie Mellon University, Pittsburgh, PA) on the basis of better attachment to plastic dishes (Miller and Williams, 1987). The HeLa-JW cells stably expressing YFP-Paxillin are described in Paran et al., 2006. HeLa-JW, B16 melanoma and rat REF52 fibroblast cells stably expressing GFP-integrin-β3, osteosarcoma U2OS, fibrosarcoma HT1080 and human dermal fibroblast (HDF) cells were cultured in Dulbecco's modified Eagle's medium (DMEM) supplemented with 10% fetal bovine serum (FBS), 1 mM sodium pyruvate and 100 U/ml penicillin-streptomycin in 5% CO2 incubator at 37°C. Primary murine cardiac fibroblast cells were a gift from Lingling Zhang (Prof. Eldad Tzahor's lab, Weizmann Institute of Science). To describe the isolation process briefly, primary cardiac cells were isolated from adult ICR mice using a neonatal dissociation kit (gentleMACS, Miltenyi Biotec) according to the manufacturer's instructions, and cultured in gelatin-coated wells (0.02%, G1393, Sigma-Aldrich) with DMEM/F12 medium supplemented with L-glutamine, Na-pyruvate, non-essential amino acids, penicillin, streptomycin, 5% horse serum and 10% FBS at 37°C and 5% CO_2_. The cell culture reagents were purchased from Biological Industries, Ltd. (Beit Haemek, Israel), and used according to the manufacturer's instructions, unless otherwise stated. No cell lines used in this study were found in the database of commonly misidentified cell lines that is maintained by ICLAC and NCBI Biosample. We did not attempt to authenticate them.

A YFP-tagged paxillin construct in pEYFP vector (Zaidel-Bar et al., 2007) was used to derive HeLa-JW cells stably expressing paxillin, kindly provided to us by Dr S. W. Katz. The cells were also transiently transfected with the following DNA plasmids: tdTomato-F-tractin (Schell et al., 2001) (a gift from M. J. Schell, Uniformed Services University, Bethesda, MA), myosin II regulatory light chain MRLC-GFP (Kengyel et al., 2010) (a gift from Drs W. Wolf and R. Chisholm, Northwestern University, Chicago, IL), mDia2 ΔDAD-GFP cloned by Dr N. O. Alieva in A.B.’s lab (Alieva et al., 2019), mCherry-VASP and mCherry-talin (M. Davidson collection in Florida State University via Dr P. Kanchawong from the Mechanobiology Institute in Singapore). All the transfections were done using Lipofectamine 2000 (Invitrogen™) following the manufacturer's protocols.

### Transfection of siRNA

Cells were seeded into 35 mm dishes on day 0 and transfected with 20 μM of Rac1, Cdc42, FMNL2, mDia2 or Arp2 siRNA (Dharmacon, ON-TARGET plus SMART pool siRNA, catalog no. L-011195-00-0005) by using Lipofectamine RNAiMAX (Invitrogen) on day 1 and day 2. Control cells were transfected with scrambled control siRNA (Dharmacon, ON-TARGET plus Non-targeting pool siRNA, catalog no. D-001810-10). Cells were imaged on day 4. The four siRNA sequences each in the smart pool were as follows. siCDC42 (M-005057-01-0005): 5′-GGAGAACCAUAUACUCUUG-3′ (siRNA1), 5′-GAUUACGACCGCUGAGUUA-3′ (siRNA2), 5′-GAUGACCCCUCUACUAUUG-3′, 5′-CGGAAUAUGUACCGACUGU-3′; siRac1 (M-003560-06-0005): 5′-UAAGGAGAUUGGUGCUGUA-3′ (siRNA1), 5′-UAAAGACACGAUCGAGAAA-3′ (siRNA2), 5′-CGGCACCACUGUCCCAACA-3′, 5′-AUGAAAGUGUCACGGGUAA-3′; siDIAPH3 (M-018997-01-0005): 5′-GAUCAGACCUCAUGAAAUG-3′ (siRNA1), 5′-GAGAAGAAAUCGAUUAAGA-3′ (siRNA2), 5′-GUAUGCAGCUCAUCAAUGC-3′, 5′-GUAGACAUUUGCAUAGAUC-3′; siArp2 (M-012076-01-0005): 5′-GAAGUUAACUACCCUAUGG-3′ (siRNA1), 5′-GCAAGUGAAUUACGAUCAA-3′ (siRNA2), 5′-GAAACGGUUCGCAUGAUUA-3′, 5′-UGGUGUGACUGUUCGAUAA-3′.

### Substrate coating

Bacterially expressed recombinant galectin-8 was purified as previously described (Hadari et al., 1995). α-Lactose-Agarose beads used for galectin-8 purification were purchased from Sigma (catalog no. L7634). Galectin-8 mutated forms were generated as previously described (Levy et al., 2006). For some experiments, galectin-8 was labeled with Alexa Fluor 568 (Alexa Fluor 568 Protein Labeling Kit, Molecular Probes, Thermo Fisher Scientific) according to the manufacturer's instructions. Fibronectin Solution (Bovine) at 1 mg/ml was purchased from Biological Industries (03-090-1-01). Fibronectin HiLyte488™ was purchased from ENDO scientific services, Israel (catalog no. FNR02-A).

Glass-bottomed Petri dishes (MatTek, P35G-1.5-14-C) were coated with 25 µg/ml galectin-8 or fibronectin solution in PBS or their mixture prepared by gentle pipetting in an Eppendorf microtube (1.5 ml). Dishes were incubated with protein solution for 2 h at room temperature and washed five times with PBS. In special control experiments with fluorescently labeled fibronectin and galectin-8, we checked that the concentration of both proteins used was saturating, so that their absorption on the glass was maximal. We chose 25 µg/ml because the absorption of the proteins onto the cover glass reaches plateau for both fibronectin and galectin-8 at this concentration. Even though the presence of 25 µg/ml galectin-8 somewhat reduced the absorption of fibronectin, at 25 µg/ml fibronectin, such reduction was minimal (Fig. S8D,E). Cells were seeded onto the protein-coated cover glass in DMEM without serum.

### Cell suspension preparation

To study the cell spreading on fibronectin and galectin-8-coated substrate, cells from 70–80% confluent cultures in 22.1 mm wells of multi-well dishes were first washed with warm PBS once, then incubated in 150 µl of trypsin-EDTA solution B (Trypsin 0.25%, EDTA 0.05%) (Biological Industries Cromwell, CT, catalog no. 03-052-1B) at 37°C for 2 min and gently suspended by addition of 5 ml serum-free DMEM with trypsin inhibitor (T9003, Sigma-Aldrich) (1 mg of trypsin inhibitor per ml trypsin-EDTA solution B). The cell suspension was centrifuged at 1000 rpm for 5 min, supernatant was removed and serum-free medium was added to re-suspend the cells. Then, cells were plated onto pre-coated Petri dishes and either imaged or fixed at appropriate time points.

### Drug treatment

The Rho activator CN03 (Cytoskeleton Inc. Denver, CO; catalog no. CN03), was added at a concentration of 1 µM 30 min following cell plating onto fibronectin or galectin-8-coated substrates, and incubated 3 h more before fixation and staining. For ROCK kinase inhibition studies, the suspended cells were pretreated with 100 µM Y27632 in serum-free DMEM for 30 min at 37°C and then were allowed to attach to either fibronectin or galectin-8-coated substrate in the presence of the inhibitor. For sugar inhibition studies, 10 mM thiodigalactoside (17154, Cayman Chemical) was added to cells in suspension and incubated for 10 min at 37°C before the cells were seeded on the substrates. The linear RGD peptide (CGGGRGD, GeneCust, HY-P2219), at a final working concentration of 20 µg/ml, was used for detachment force measurements, and the cyclic RGD peptide (GRGDSPK, Sigma-Aldrich, G1269), at a final working concentration of 10 µg/ml was used for focal adhesion and cell spreading measurements. RGD was added to cell suspension 10 min before plating and remained in the medium during the experiments.

### Cryo-electron tomography

Cells were applied onto galectin-8-coated EM grids with carbon support film (R2/2, Au mesh; Quantifoil, Jena, Germany). After 20 min incubation, a 4 µl drop of fiducial gold marker (10 nm; Aurion, Wageningen, The Netherlands) was added to the sample prior to plunge freezing into liquid ethane. A Titan Krios transmission electron microscope (Thermo Fisher Scientific, Waltham, MA) equipped with a Quantum energy filter and a K2-Summit direct electron detector (Gatan, Pleasanton, CA) was used for cryo-EM data acquisition. The microscope was operated at 300 keV in zero-loss mode with the energy filter slit width set to 20 eV.

The tomograms were recorded with an electron flux of ∼10 electrons per pixel/s using SerialEM (Mastronarde, 2005). Tilt series were acquired at a magnification of 42,000×, and a dose-fractionated frame rate of six frames per 1.2 s. The tilt-series covered an angular range between −60° and +60°, and were recorded with tilt increments of 2° and a defocus of −4 μm. The accumulated electron dose did not exceed ∼120 e^−^/Å^2^. Finally, the structures were reconstructed using IMOD.

### Cell adhesion forces

Two defined regions of the glass-bottomed Petri dish (GWSB-5030, WillCo Wells) were first freshly coated with galectin and fibronectin, respectively, as explained above. Adhesion forces of cells to the underlying substrate for a contact time of 5 min were measured using Single Cell Force Spectroscopy (SCFS) with FluidFM^®^ technology (Cytosurge, Switzerland) incorporated to an Atomic Force Microscope (AFM) Flex-FPM system (Nanosurf, Germany) (Guillaume-Gentil et al., 2014). The system was mounted on an Axio Observer Z1 inverted microscope (Carl Zeiss, Germany) for the visualization of the cells. Micropipette cantilevers (Cytosurge, Switzerland) with an aperture of 4 µm in diameter and 0.3 N/m nominal spring constant were used. A cell was immobilized at the tip of the cantilever by applying a soft negative pressure, and then brought into contact with the corresponding substrate by approaching at a speed of 1 µm/s until reaching a set point of 5 nN. This force was kept constant during the 5 min the cell was kept in contact with the test material. After this time, the cantilever holding the cell was retracted from the surface and its deflection during the retraction was recorded (Sancho et al., 2017). The deflection of the cantilever is directly proportional to the force exerted by the cells against the substrate while they are being pulled away from it, and the maximum force peak is used as the indicator of cell adhesion force (Potthoff et al., 2012). Ten individual cells were measured under each experimental condition.

### Immunofluorescence staining

For immunostaining, cells cultured on glass-bottomed dishes were fixed and permeabilized in phosphate-buffered saline (PBS) containing 0.25% Triton X-100, 0.25% glutaraldehyde and 3% paraformaldehyde at 37°C for 15 min. The cells were then washed twice with PBS for 10 min each. Before staining, the fixed cells were treated with 1 mg/ml NaBH_4_ in PBS on ice for 15 min. Then, cells were washed with PBS, incubated with blocking solution (5% bovine serum albumin in PBS) for 1 h at room temperature and washed with PBS again. Then, cells were incubated with appropriate primary antibodies (anti-paxillin, anti-myosin IIA, anti-phosphotyrosine, anti-integrin and anti-vinculin) at room temperature for 1 h and, after three times washing with PBS for 10 min, with appropriate fluorescently labeled secondary antibody and phalloidin to visualize actin. Goat anti-rabbit and goat anti-Mouse IgG (H+L) Cross-Adsorbed ReadyProbes™ secondary antibody were purchased from Thermo Fisher (catalog no. R37116 and R37114). Rabbit IgG-Alexa Fluor 488 and mouse IgG-Alexa Fluor 647 secondary antibodies were purchased from Thermo Fisher (catalog no. A21245 and A32728, respectively), and used at dilution 1:400. Phalloidin–tetramethylrhodamine B isothiocyanate was obtained from Sigma-Aldrich (catalog no. P1951) and was used at 1:400 dilution. Purified mouse anti-paxillin (BD Transduction Laboratories, clone 349, catalog no. 610052) was used at dilution 1:200, anti-myosin IIA, non-muscle antibody produced in rabbit (Sigma, catalog no. M8064) at dilution 1:400. Mouse anti-human β1 integrin (BD Bio-science, clone HUTS22; catalog no. 556048) monoclonal antibody was used to recognize the extended conformation of integrin β1 (high affinity for ligand, termed ‘active’), and was used at 1:100 dilution. The monoclonal antibody against mouse anti-human integrin β1, recognizing all β1 integrin species, was P5D2 (Developmental Studies Hybridoma Bank; 1:10 dilution). Monoclonal human vinculin, phosphotyrosine and polyclonal zyxin antibodies were prepared by the Antibody Production Laboratory of the Department of Biological Services, Weizmann Institute of Science and used at 1:50 dilution.

### Microscopy and live cell imaging

Cells were plated at a density of 5×10^4^ cells ml^−1^ onto the 35 mm cell culture dish with 14 mm-diameter glass bottom (MatTek, catalog no. P35G-1.5-14-C) coated with fibronectin, galectin-8 or their mixture as described above. Cells were imaged in medium with low level of background fluorescence FluoroBrite DMEM (Thermo Fisher, catalog no. A1896701). Video recordings started 5 min after the cells had been added to the dish. Differential interference contrast (DIC) and interference reflection microscopy (IRM) time-lapse imaging were carried out using the DeltaVision RT microscopy system (Applied Precision Inc., Issaquah, WA), equipped with a 60× oil immersion objective (1.40 NA, UPlanSApo), or 100× oil immersion objective (1.3 NA, UPlanSApo), at time intervals of 2 s or 10 s between frames. Total internal reflection fluorescent (TIRF) images were acquired using the DeltaVision Elite microscopy system equipped with a multi-line TIRF module (Applied Precision, Inc.), and time-lapse movies were taken at 30-s intervals, unless otherwise indicated. Super-resolution SIM imaging was performed using W1-spinning-disk confocal unit coupled with the live super-resolution (SR) module [spinning disk based structured illumination super resolution (York et al., 2013)] (GatacaSystems), mounted on Eclipse microscope with Perfect Focus System, supplemented with the objective Plan Apo 100× oil NA1.45 and scientific complementary metal–oxide–semiconductor (sCMOS) camera Prime95B (Photometrics). Confocal images and videos were taken with ANDOR Dragonfly spinning disk confocal microscope using 100× objective and an sCMOS (Zyla) camera.

### Image analysis

#### Projected cell area measurements

IRM images of cells taken at different time intervals after plating were first subtracted using a background image taken before plating the cell. Intensity thresholding by using the method described by Otsu (1979) was then applied to segment the cell. After cell segmentation, individual objects were identified, and the object with the largest area was preserved and considered as the main cell body. To measure the projected cell area of fixed cells, we applied the same algorithm to actin fluorescence images instead of IRM images. The measurements were implemented using Matlab and the analysis were performed automatically

#### Filopodia measurements

To identify the filopodia and quantify their length and number in IRM and actin fluorescence images, we used the FiloDetect algorithm (Nilufar et al., 2013). Filopodia were defined as high-aspect ratio (≥1.5:1) objects protruding from the ‘main cell body’ with a smooth boundary. The filopodia shorter than 0.6 µm were ignored. The algorithms were implemented using Matlab and the analyses were performed automatically.

#### Myosin II filament measurements

Live-cell recording of myosin II was performed using SIM imaging. A thresholding algorithm was first applied to actin images to segment the cell from the background. Then, cells were segmented into 1-pixel-width rings with the same contour as the cell edge without filopodia. The average GFP-MRLC intensity was calculated for each ring. The measurements were implemented using Matlab and analyses were performed automatically.

#### Paxillin structure measurements

Ilastik software (Berg et al., 2019) was applied to images stained for paxillin. The software runs a random forest classifier (Criminisi et al., 2011) to the images to segment paxillin from the background. After the segmentation using Ilastik software, the fluorescent intensity and area were analyzed automatically with Matlab.

#### Kymograph generation

Elongated rectangular areas perpendicular to the cell edge, with widths between 200 nm and 3 μm and a length of 10–20 μm, were selected in the movies and put side by side representing corresponding time points using ImageJ built-in montage function.

Calculation of *P* values was performed using two-tailed Mann–Whitney test for Fig. 5. For all other *P* values, two-sample two-tailed *t*-test was performed. All the tests were performed using OriginLab software.

Box and whisker plots show median values (middle line inside the box), upper and lower quartiles (upper and lower bound of the box), maximum and minimum (upper and lower cap), and outliers (whiskers) – i.e. values that are 1.5× larger than the upper or 1.5× smaller than the lower quartiles). *P<0.05, **P<0.01, ***P<0.005, ****P<0.001, n.s., not significant.

## Supplementary Material

Supplementary information

Reviewer comments
