# Supplementary material for: Differential cellular responses to adhesive interactions with galectin-8- and fibronectin-coated substrates
Source: J Cell Sci. 2021 Apr 27;134(8):jcs252221. doi: 10.1242/jcs.252221 (PMC8106957; doi:10.1242/jcs.252221)
Supplement: Supplementary information [file joces-134-252221-s1.pdf]

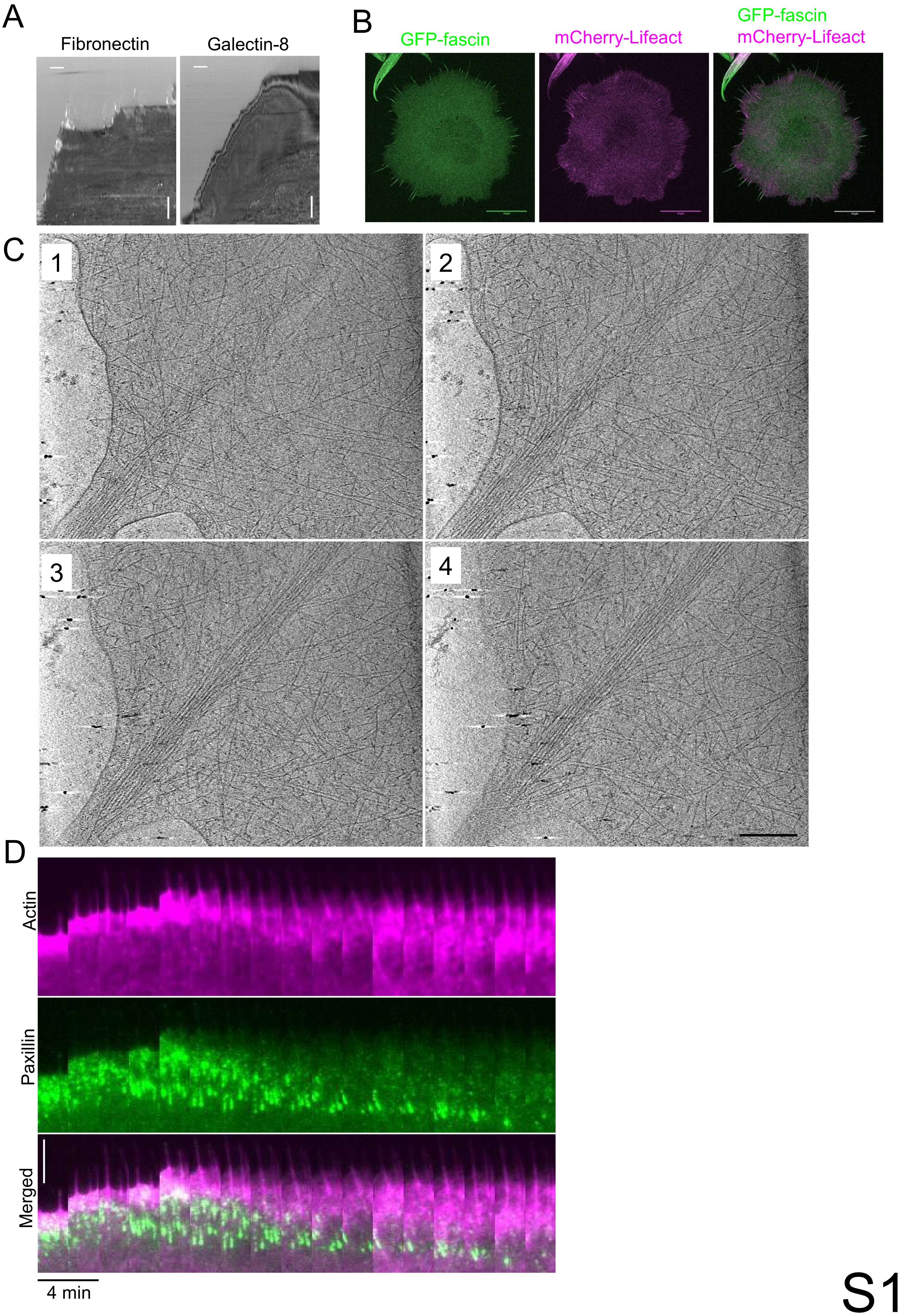
A
Fibronectin
Galectin-8
B
GFP-fascin
mCherry-Lifeact
GFP-fascin
mCherry-Lifeact
C
1
2
3
4
D
Actin
Paxillin
Merged
4 min

S1

Figure S1. Lamellipodia, filopodia and paxillin adhesions in HeLa cells, plated on galectin-8 coated substrates.

A. Typical kymographs depicting the process of cell spreading on fibronectin and galectin-8. Images of one pixel-wide strip oriented perpendicular to the cell edge were taken every 10 seconds and mounted along the horizontal time axis. The temporal horizontal scale bar represents 100 seconds, and the vertical spatial scale bar represents 2 µm.

B. Cells transfected with GFP-fascin and mCherry-Lifeact plated on galectin-8 coated substrates and fixed 20 minutes following plating. Note that filopodia contain fascin. Scale bar: 15 µm.

C. Cryo-electron tomography of a HeLa cell spreading on an EM grid with carbon support film, coated with galectin-8. The cells were incubated for 20 minutes at 37ºC and processed for cryo-electron tomography as described in the Materials and Methods section. The images (1 - 4) display four slices, 4 nm-thick each and 13 nm apart. The bottom slice (1) is located 28 nm above the support. Scale bar: 200 nm

D. Kymograph depicting the dynamics of lamellipodia and paxillin clusters in cells plated on galectin-8. The cell was labeled with tdTomato-F-tractin and YFP-paxillin, and imaged 20 minutes after plating on a galectin-8 coated substrate. The rectangular stripe perpendicular to the cell’s leading edge was acquired every 0.5 minutes. Note the formation of numerous paxillin clusters associated with actin-rich lamellipodia, which moves centripetally and eventually disappeared. Vertical scale bar: 5 µm. The time interval between the frames is 2 minutes, and the total duration of the kymograph is 34 minutes. These results are based on three independent experiments

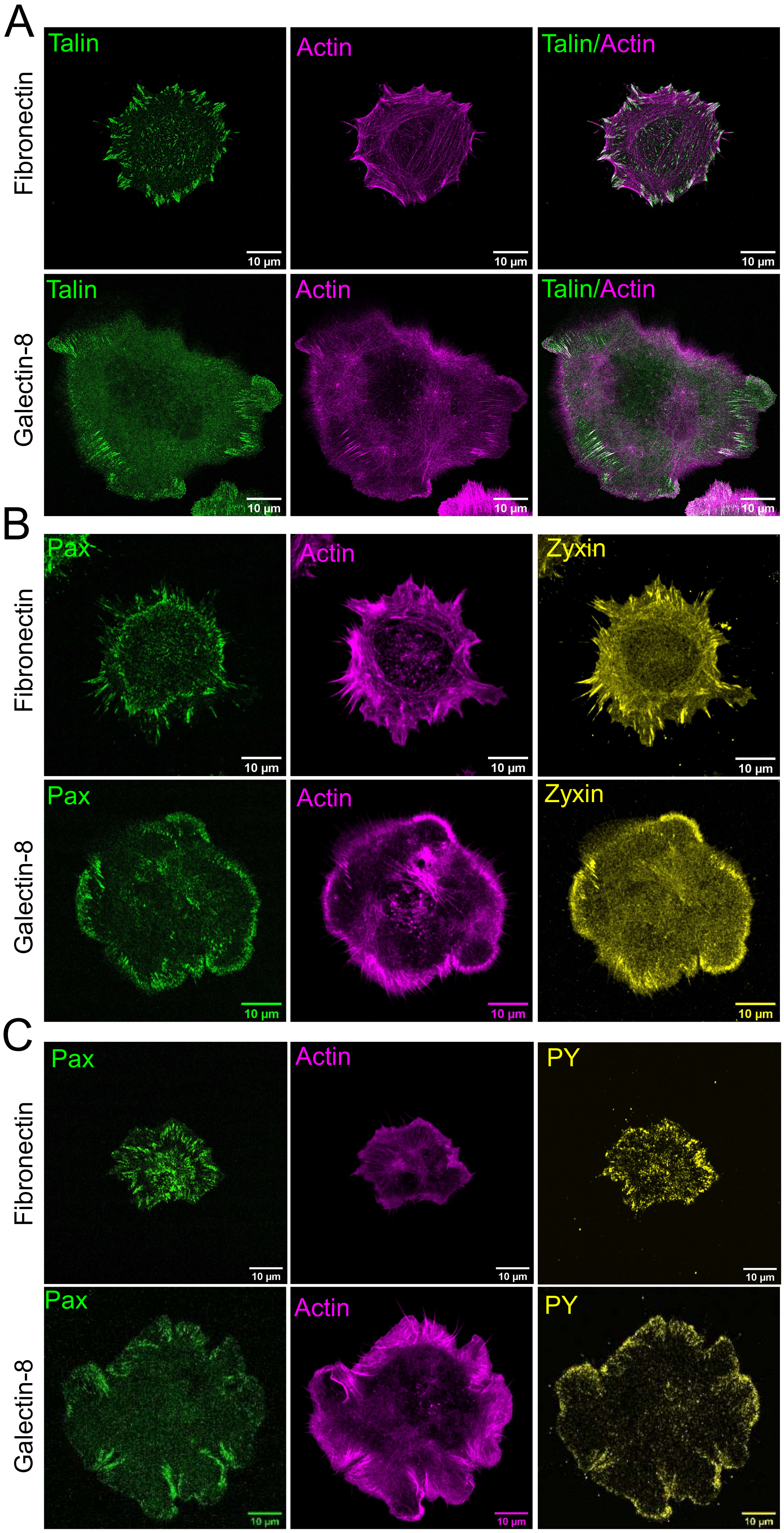
A
Talin
Actin
Talin/Actin
Fibronectin
Galectin-8
10 µm
B
Pax
Actin
Zyxin
C
PY

S2

Figure S2. **Paxillin containing clusters in HelaJW cells on galectin-8 coated substrates associated with actin and are talin-, zyxin-, and phosphotyrosine- positive.**

A. HelaJW cells were transfected with GFP-talin together with mCherry-lifeact, and plated on fibronectin and galectin-8 coated substrates for 30 minutes before fixation. Talin formed classical focal adhesions on fibronectin (upper row), and peripheral clusters similar to those formed by paxillin on galectin-8 (lower row).

B. C. HelaJW cells stably expressing YFP-paxillin were plated on fibronectin (upper rows) or galectin-8 (lower rows) for 30 minutes before fixation and staining with phalloidin and antibodies against either zyxin (B) and phosphotyrosine (PY) (C). Both zyxin and phosphotyrosine co-localize with paxillin-positive structures on fibronectin- and galectin-8- coated substrates. Scale bar: 10 µm.

Journal of Cell Science • Supplementary information

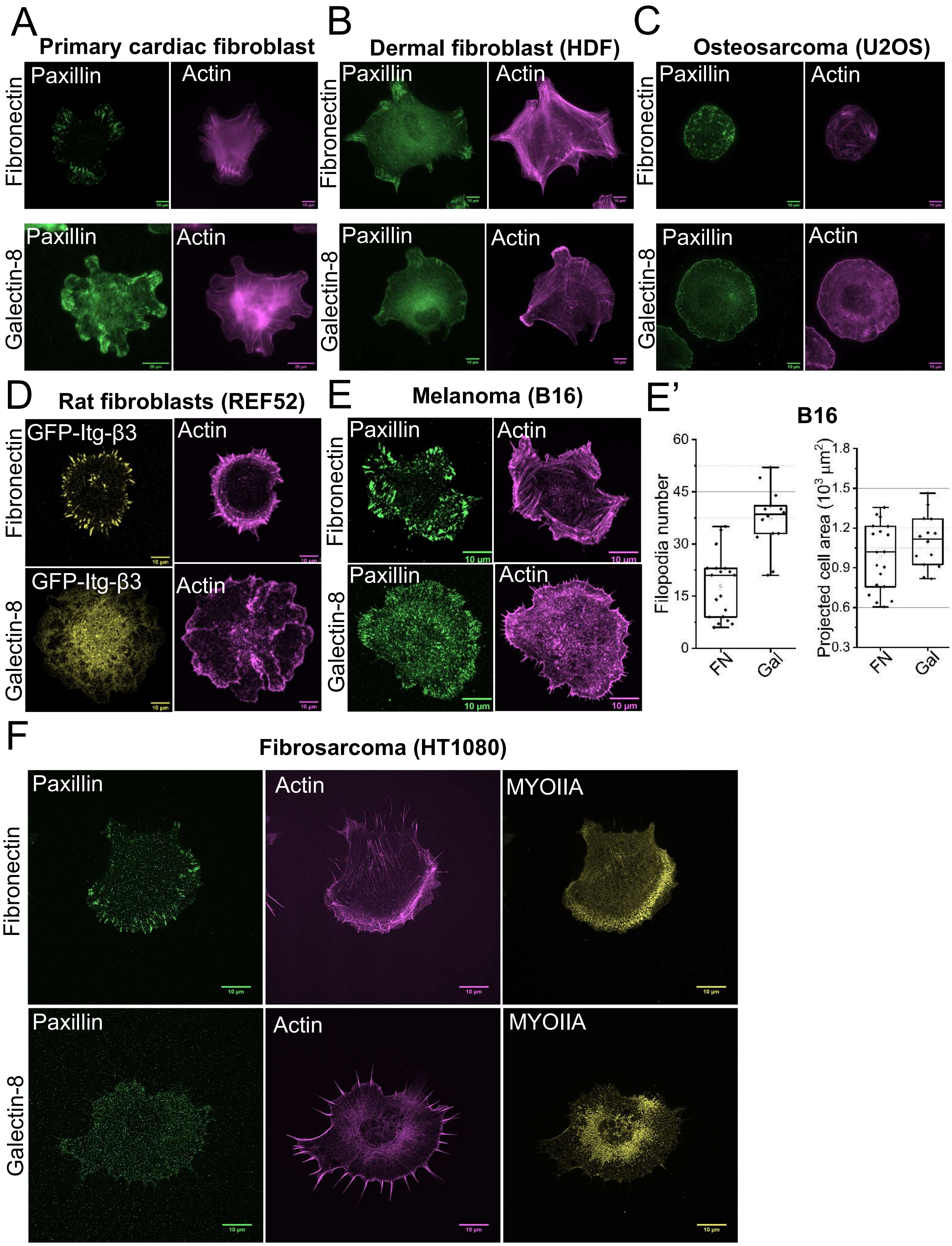
A
Primary cardiac fibroblast
B
Dermal fibroblast (HDF)
C
Osteosarcoma (U2OS)
Fibronectin
Galectin-8
Paxillin
Actin
D
Rat fibroblasts (REF52)
GFP-Itg-β3
E
Melanoma (B16)
E'
B16
Filopodia number
Projected cell area (10³ μm²)
FN
Gal
F
Fibrosarcoma (HT1080)
MYOIIA

Figure S3: **Various cell types demonstrate distinct actin cytoskeleton and adhesion organization on fibronectin and galectin-8, similar to those formed in HeLa cells.**

The cells tested include primary murine cardiac fibroblasts (A), human dermal fibroblasts (HDF) (B), human osteosarcoma cells (U2OS) (C), rat embryo fibroblasts (REF-52) (D), mouse melanoma cells (B16) (E), and human fibrosarcoma cells (HT1080) (F). Cells were fixed half an hour following plating in serum-free medium on either fibronectin or galectin-8 coated substrates. REF52 and B16 cells stably express GFP-β3-integrin. After fixation, the cells were stained with phalloidin to visualize F-actin, and with antibodies against paxillin (A, B, C, E, F) and myosin-IIA (F). The box and whiskers plots presenting the projected cell areas and filopodia numbers in B16 cells plated on fibronectin and galectin-8 are shown in E, and these results are based on three independent experiments

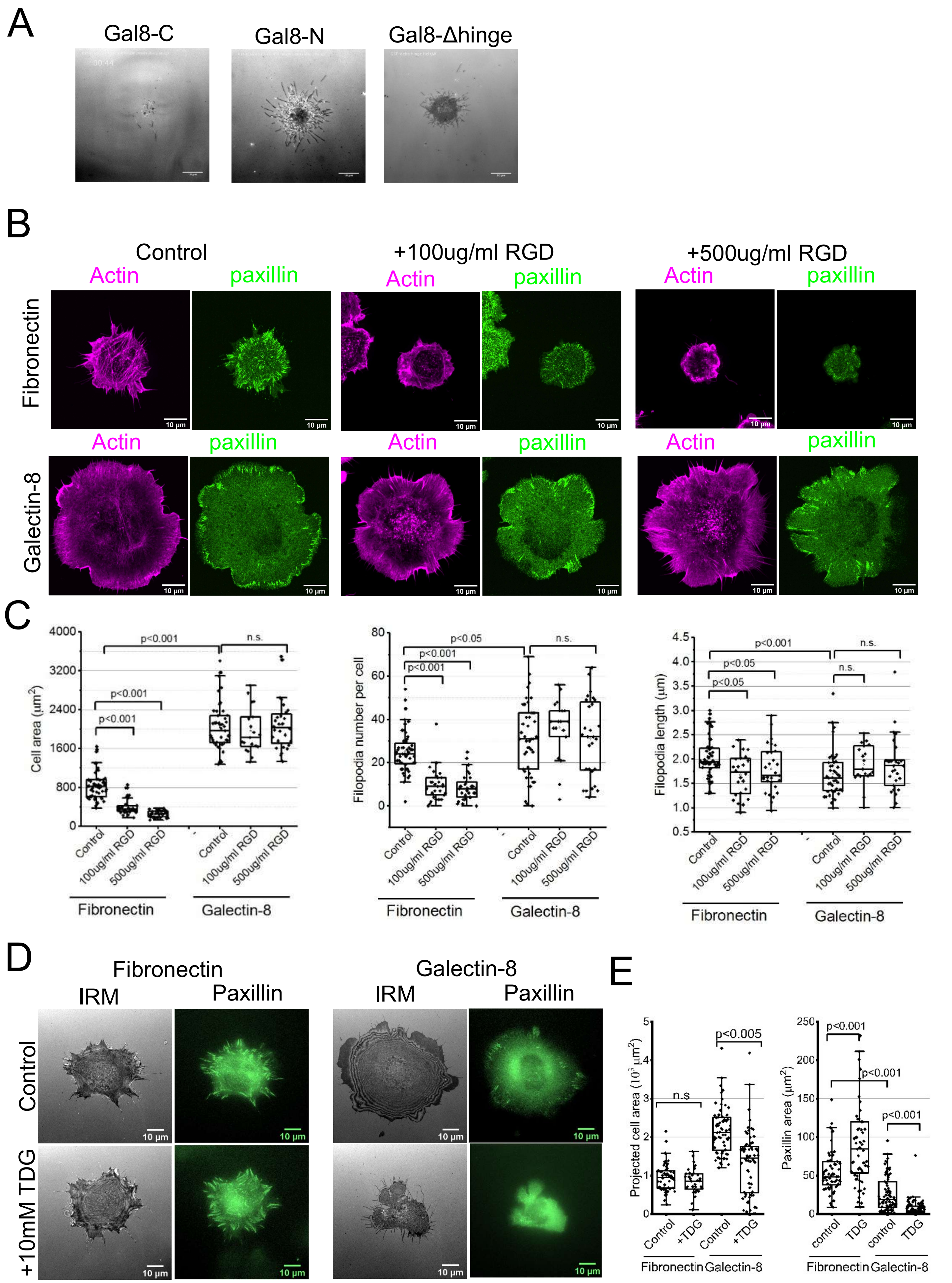

S4

Journal of Cell Science • Supplementary information

Figure S4: **Molecular requirements for cell spreading on fibronectin and galectin-8**

A. Cell spreading on substrates coated with different truncated galectin-8 mutants visualized by interference reflection microscopy.

B. Cyclic RGD peptide inhibits cell spreading and formation of paxillin positive structures on fibronectin but not on galectin-8**.** Cells stably expressing YFP-paxillin were fixed 20 minutes following plating on fibronectin and galectin-8 in the absence or presence of 100 µg/ml or 500 µg/ml cyclic RGD, and were then stained with phalloidin to visualize actin.

C. Box and whiskers plots showing projected cell area (left), filopodia number (middle) and filopodia length (right) in cells spreading on fibronectin or galectin-8 with or without RGD. *p* values were calculated using two-tailed t-test. These results are based on three independent experiments

D. Thiodigalactoside (TDG) inhibits cell spreading on galectin-8 but not on fibronectin coated substrates. IRM and YFP-paxillin images of cells spreading for half an hour on fibronectin or on galectin-8 without or with 10mM TDG.

E. Box and whiskers plots showing projected cell area (left) and paxillin area (right) in cells spreading on fibronectin or galectin-8 with or without TDG. *p* values were calculated using two-tailed t-test.

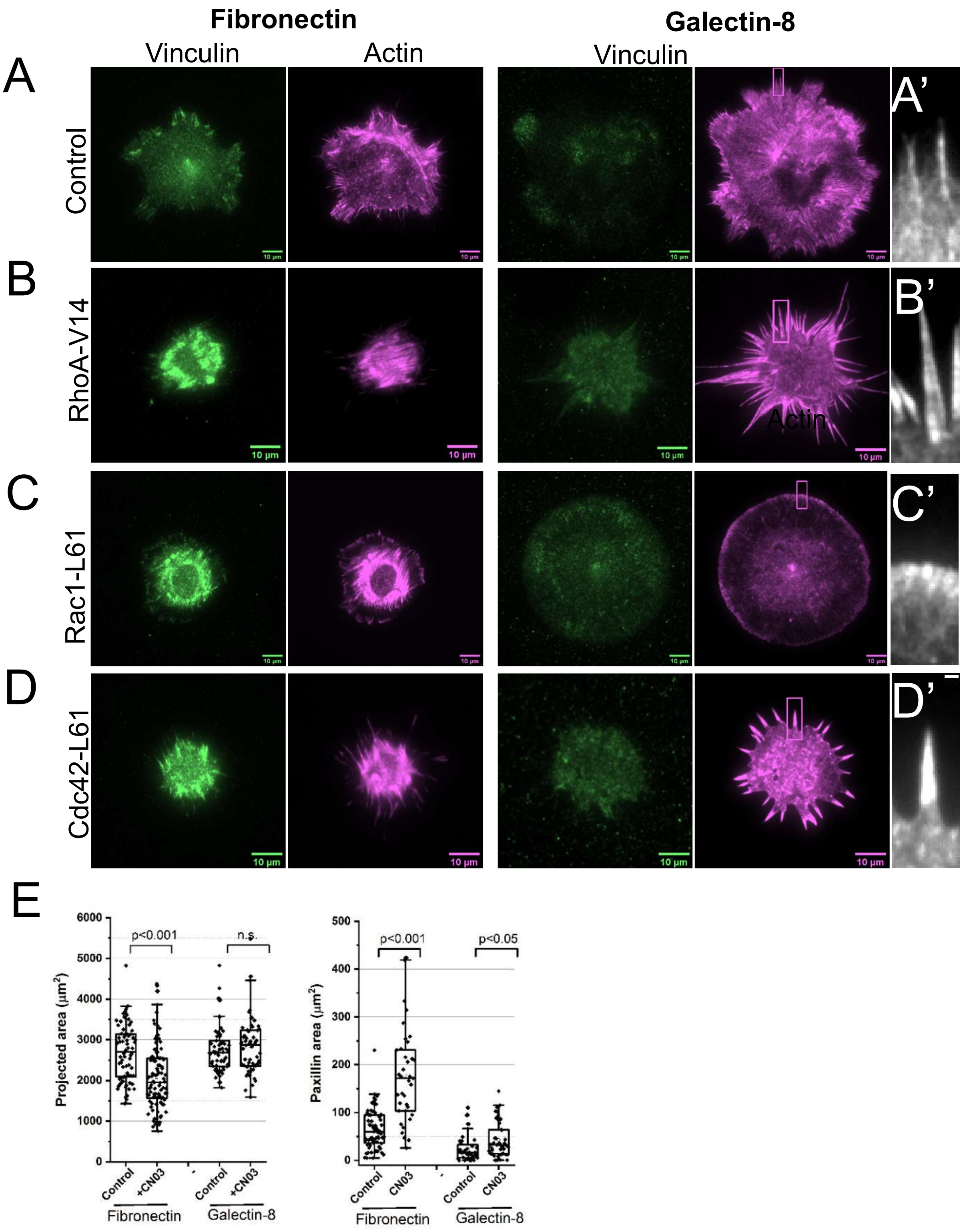

Journal of Cell Science • Supplementary information

Figure S5: **Effects of small Rho GTPases on cell spreading on fibronectin- and galectin-8 coated substrates.**

(**A-D**) Paxillin-positive adhesions and F-actin in control cells (A) and cells expressing constitutively active RhoA-V14 (B), Rac1-L61(C), and Cdc42-L61(D), 2 hours following plating on fibronectin- or galectin-8- coated substrates. Cells were fixed and stained with paxillin antibody and TRITC-phalloidin to visualize F-actin. Scale bars: 10 μm. Boxed areas at high magnification are shown in A', B', C', D', respectively. Scale bar shown in F': 2 μm**.**

(**E**) Effects of pharmacological RhoA activation on formation of paxillin-positive adhesions and actin cytoskeleton structures in cells spreading on fibronectin and galectin-8 substrates. Cells were plated on fibronectin- or galectin-8 coated substrates in the absence or presence of 1 μM RhoA activator CN03, and fixed 3 hours following plating. Box and whiskers plots showing projected cell area and total area of paxillin clusters per cell. Morphometric measurements and presentation of results were performed as described in the legend to Figure 6. These results are based on three independent experiments

Journal of Cell Science • Supplementary information

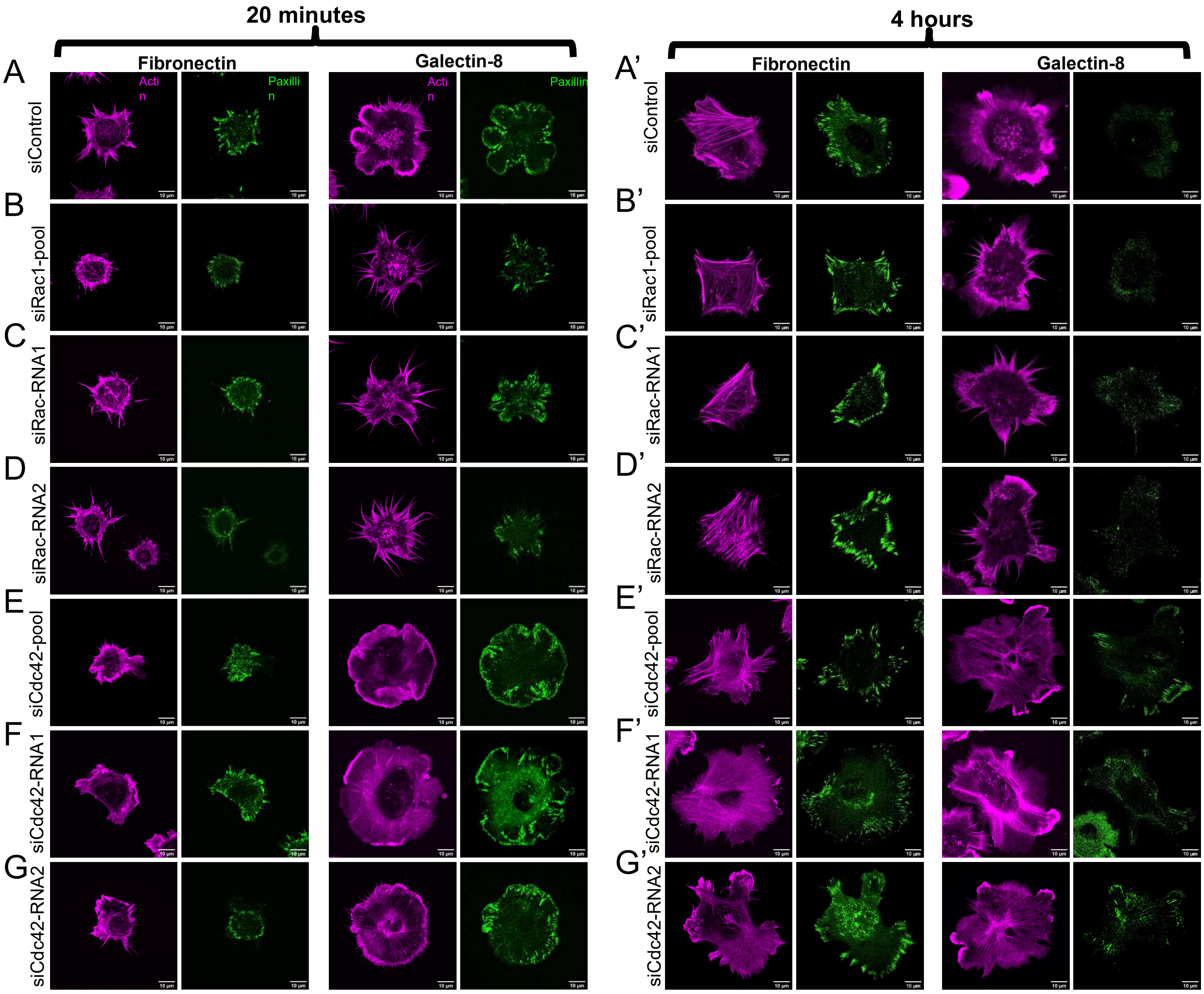

Figure S6: Effects of depletion of small Rho GTPases on cell spreading on fibronectin and galectin-8 coated substrates 20 minutes and 4 hours following cell plating.

Cells transfected with control siRNA (A), siRNA for Rac1 (B-D) and for Cdc42 (E-G) were plated on fibronectin and galectin-8 coated substrates, and fixed 20 minutes later (left panel) or 4 hours later (right panel). YFP-paxillin (green) stably expressing cells were then stained with TRITC-phalloidin to visualize F-actin (magenta). Scale bars: 10 µm. Three individual siRNA reagents were used to exclude possible off-target effects: siRNA-smartpool (Dharmacon) containing four individual siRNAs, and two individual siRNAs (# 1 and 2). The corresponding siRNA sequences are presented in the Materials and Methods.

Journal of Cell Science • Supplementary information

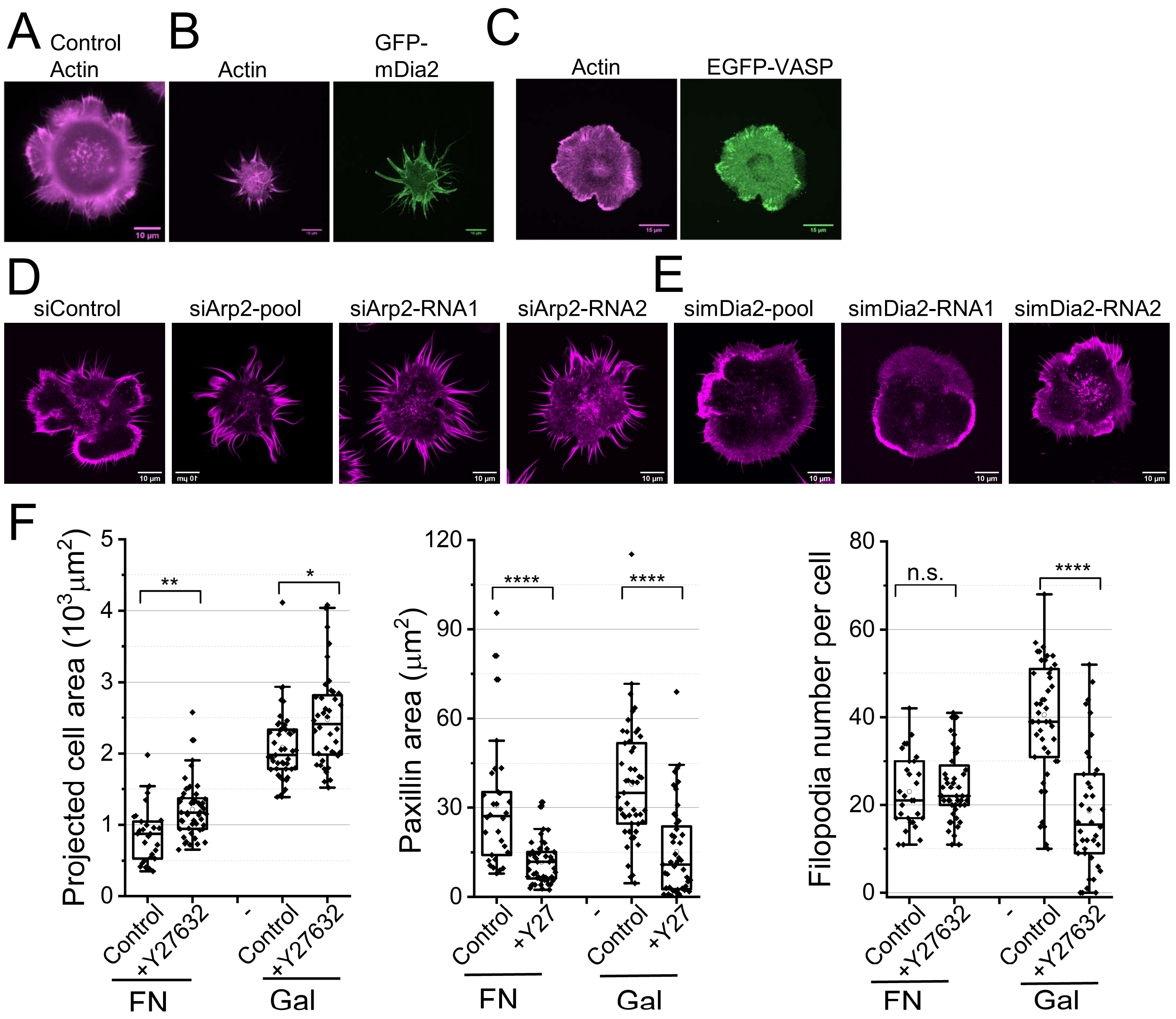

Figure S7: **Effects of downstream effectors of Rho family G-proteins on cell spreading on galectin-8 coated substrates.**

A-E. Control cells (A), cells expressing the constitutively active formins EGFP-mDia2 (B), cells over-expressing actin polymerization activator EGFP-VASP (C), as well as cells expressing siRNAs to Arp2 (D) and mDia2 (E) were plated on a galectin-8 coated substrate, fixed 20 minutes following plating, and stained with TRITC-phalloidin to visualize F-actin. In (B) and (C), actin images (left) and images showing the localization of EGFP-mDia2 and EGFP-VASP in the same cells (right) are shown. D. E. shows the images of actin staining.

F. Effects of inhibition of Rho kinase (ROCK) on cell spreading on fibronectin and galectin-8 substrates. Cells were plated on fibronectin and galectin-8 substrates in the absence (control) and the presence of 100 μM of Y27632 (Y27), and fixed 30 minutes after plating. Projected cell areas, total areas of paxillin clusters per cell, and filopodia number were measured. Morphometric measurements and presentation of results were performed as described in the legend to Figure 5 and 6. Two tailed t-test was performed, and N ≥30 cells were assessed under each experimental condition. These results are based on three independent experiments

Journal of Cell Science • Supplementary information

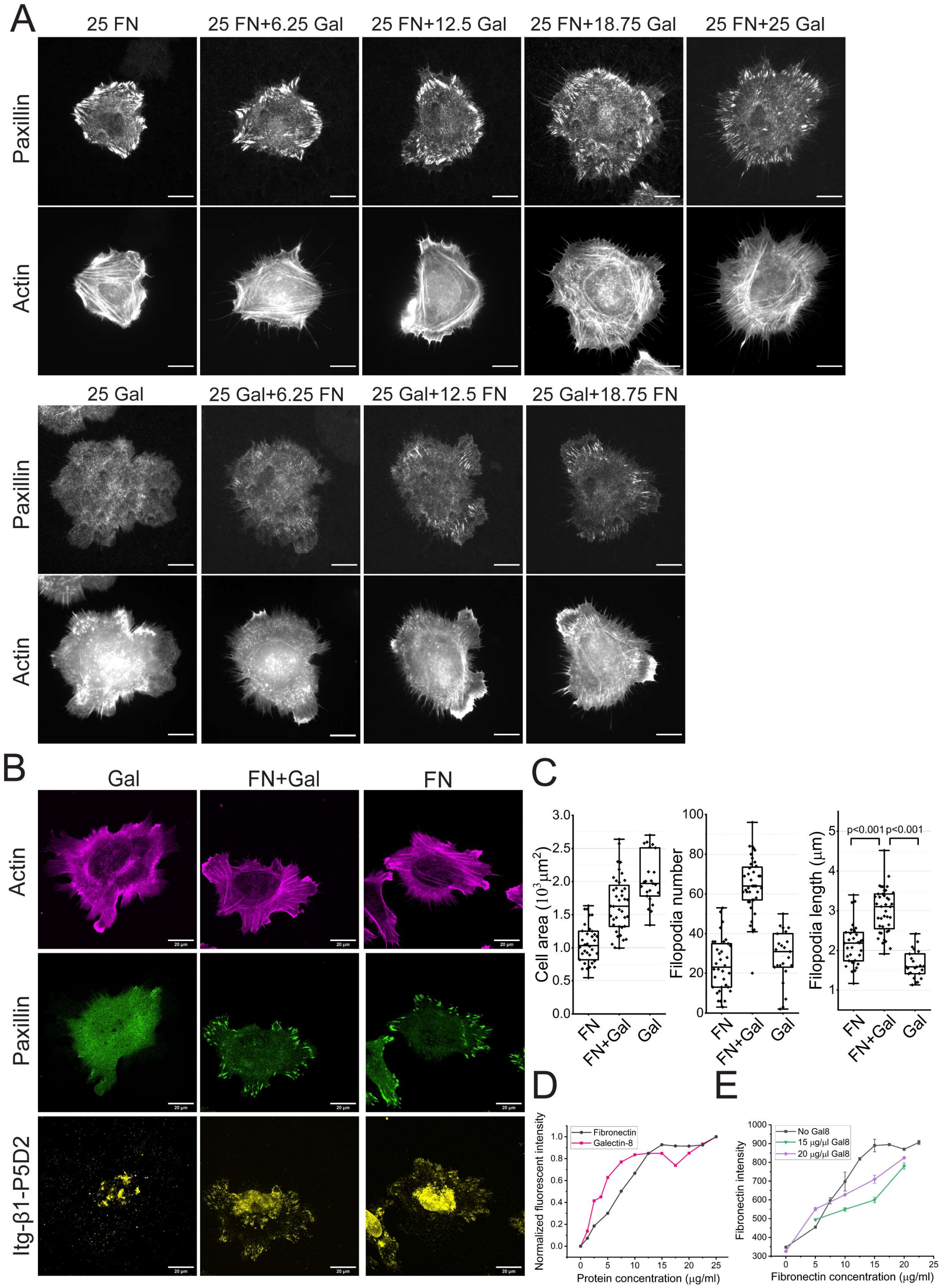

Journal of Cell Science • Supplementary information

Figure S8: **Cell spreading on composite matrices comprising fibronectin and galectin-8 at different ratios.**

A. Focal adhesions visualized by YFP-paxillin (upper row), and F-actin visualized by TRITC-phalloidin staining (lower row) in cells fixed 4 hours following plating on substrates coated with different combinations of fibronectin (FN) and galectin-8 (Gal), as indicated. Scale bar: 10 μm

B. Activated β1 integrin staining of YFP-paxillin-expressing cells, plated on 25 μg/ml fibronectin, 25 μg/ml galectin-8, or a mixture of the two at the concentration of 25 μg/ml for each. Cells were fixed at four hours following plating and stained with P5D2-s antibody against activated β1 integrin. Scale bar: 20 μm.

C. Quantification of filopodia number, cell area, and filopodia length in cells spreading for 20 minutes on 25 μg/ml fibronectin, 25 μg/ml galectin, or a mixture of the two at the concentration of 25 μg/ml for each. *p* values were calculated using two-tailed t-tests. The experiments were performed three times.

D. Fibronectin and galectin-8 reaches absorption on glass bottomed petri-dish. Note that the protein absorption on the surface reached plateau at the concentration of 15 μg/ml for both galectin-8 and fibronectin. These results are based on two independent experiments

E. Fibronectin absorption on the glass bottomed petri-dish in the presence of galectin-8. At low concentrations of fibronectin (≤15 μg/ml), the absorption of fibronectin is reduced by galectin-8. At high concentration of fibronectin (≥20 μg/ml), the addition of galectin-8 does not significantly reduce fibronectin absorptions. The experiments were performed twice.

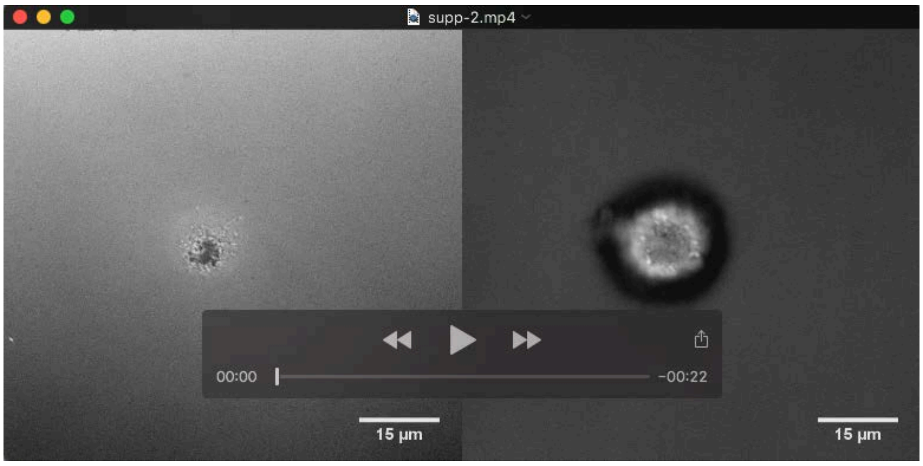

Movie 1: Spreading of cells on fibronectin coated substrates. Representative time-lapse series showing cells spreading on the fibronectin-coated substrate, imaged by interference reflection microscopy (IRM) (left) and differential interference contrast microscopy (DIC) (right). The time interval is 5 seconds. Scale bar: 15 µm. Timestamp: mm:ss, The display rate: 15 frames per second.

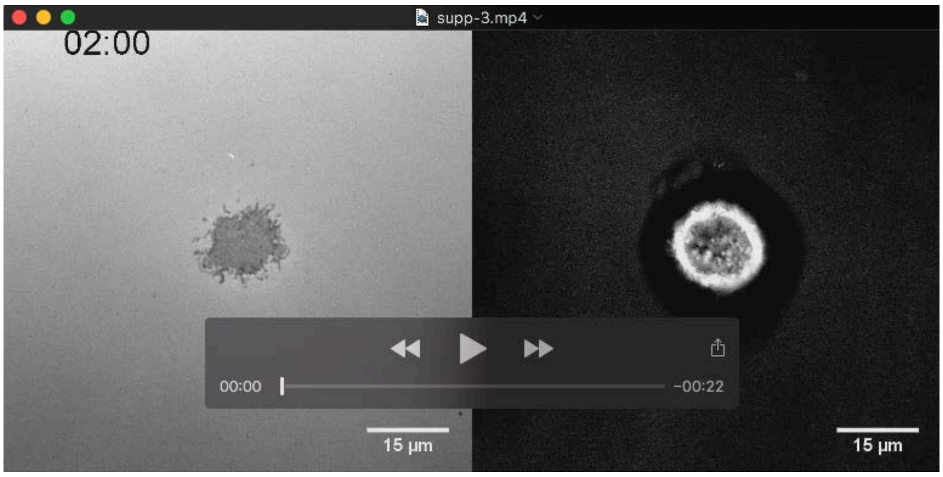

Movie 2: Spreading of cells on galectin-8 coated substrates. Representative time-lapse series showing cells spreading on galectin-8-coated substrate, imaged by interference reflection microscopy (IRM) (left) and differential interference contrast microscopy (DIC) (right). The time interval is 5 seconds. Scale bar: 15 µm. Timestamp: mm:ss, The display rate: 15 frames per second.

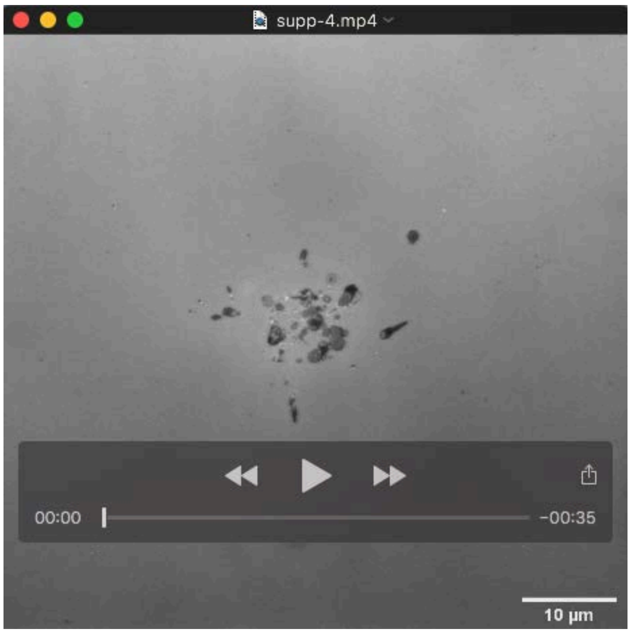

Movie 3: Spreading of cells on galectin-8 coated substrates. Representative time-lapse series showing cells spreading on galectin-8-coated substrate, imaged by interference reflection microscopy (IRM). The time interval is 2 seconds. Scale bar: 15 μm. Timestamp: mm:ss, The display rate: 15 frames per second.

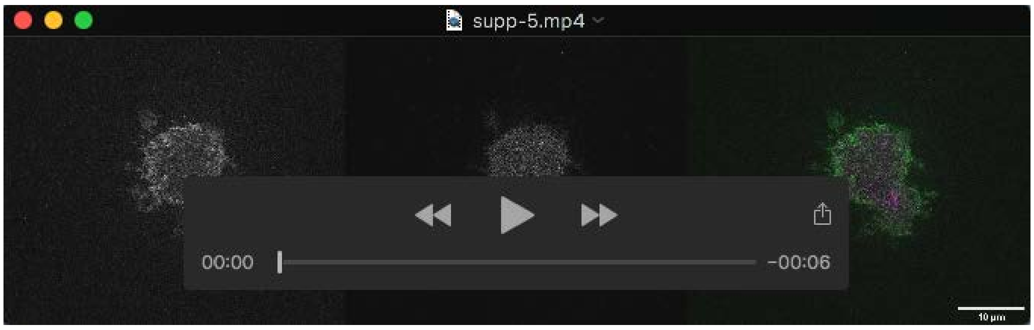

Movie 4: Actin and myosin II dynamics during cell spreading on fibronectin. Representative time-lapse series showing cells spreading on fibronectin-coated substrate, imaged by structured illumination microscopy (SIM). Cells were labeled with tdTomato-F-tractin (actin) and GFP myosin II regulatory light chain (MRLC). The time interval is 9.5 seconds. Scale bar: 10 μm. Timestamp: mm:ss, The display rate: 15 frames per second.

Journal of Cell Science • Supplementary information

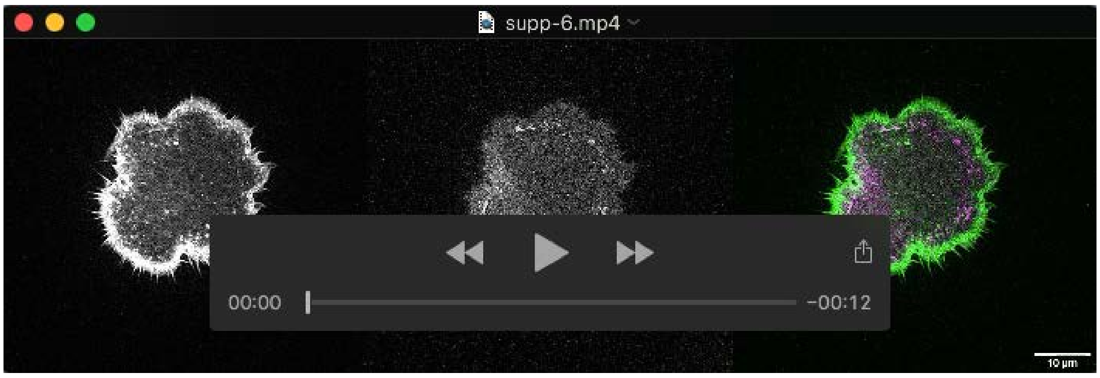

Movie 5: Actin and myosin II dynamics during cell spreading on galectin-8. Representative time-lapse series showing cells spreading on galectin-8-coated substrate, imaged by structured illumination microscopy SIM. Cells were labeled with tdTomato-F-tractin (actin) and GFP myosin II regulatory light chain (MRLC). The time interval is 10 seconds. Scale bar: 10 µm. Timestamp: mm:ss, display rate: 15 frames per second.

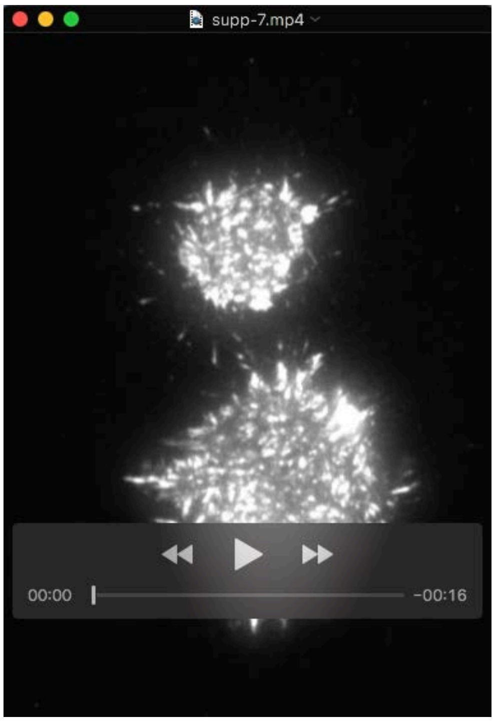

Movie 6: Paxillin dynamics during cell spreading on fibronectin. Representative time-lapse series showing cells spreading on fibronectin-coated substrate, imaged by TIRF. Cells were labeled with YFP-paxillin and tdTomato-F-tractin. The time interval is 30 seconds. Scale bar: 10 µm. Timestamp: mm:ss, display rate: 15 frames per second.

Journal of Cell Science • Supplementary information

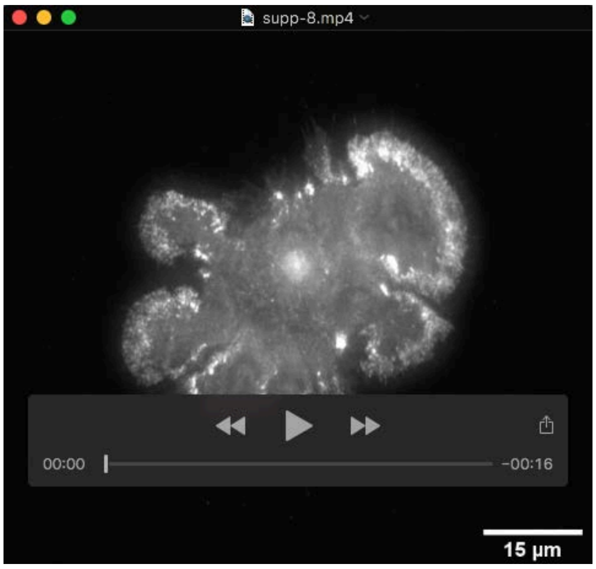

Movie 7: Paxillin dynamics during cell spreading on galectin-8. Representative time-lapse series showing cells spreading on galectin-8-coated substrate, imaged by TIRF. Cells were labeled with YFP-paxillin and tdTomato-F-tractin. The time interval is 30 seconds. Scale bar: 10 µm. Timestamp: mm:ss, the display rate: 15 frames per second.

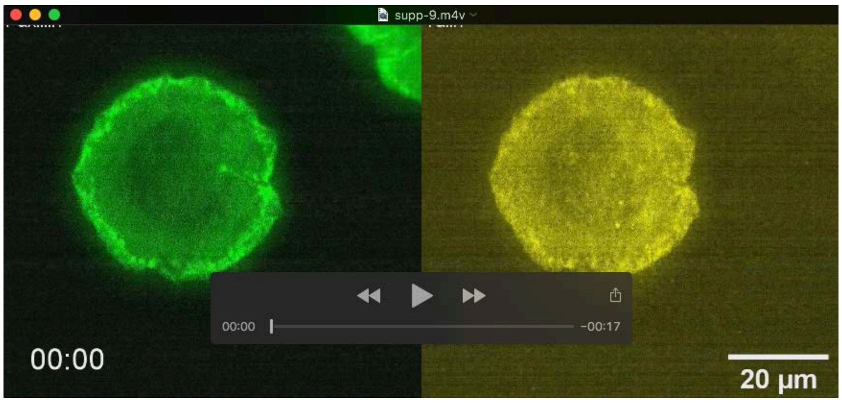

Movie 8: Talin and paxillin dynamics during cell spreading on galectin-8. Representative time-lapse series showing cells spreading on galectin-8-coated substrate, imaged by TIRF. YFP-paxillin stably expressing cells were transfected with mCherry-Talin. The time interval is 30 seconds. Scale bar: 20 µm. Timestamp: mm:ss, the display rate: 7 frames per second.

Journal of Cell Science • Supplementary information

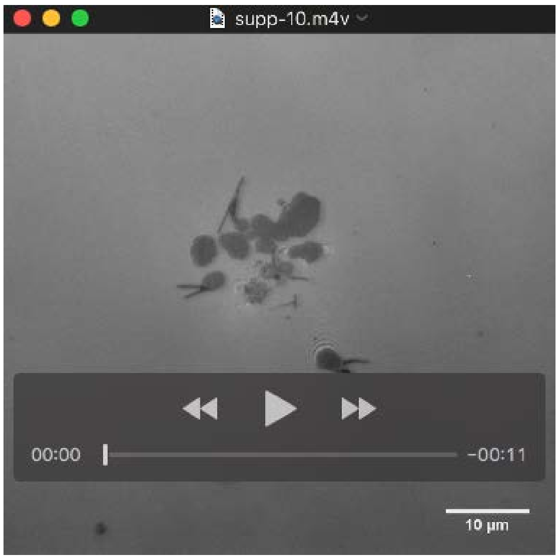

Movie 9: Spreading of cells expressing constitutively active RhoA on galectin-8 coated substrates. Representative time-lapse series showing GFP-RhoA-V14 expressing cells spreading galectin-8-coated substrate, imaged by interference reflection microscopy (IRM). The time interval is 5 seconds. Scale bar: 10 µm. Timestamp: mm:ss, the display rate: 15 frames per second.

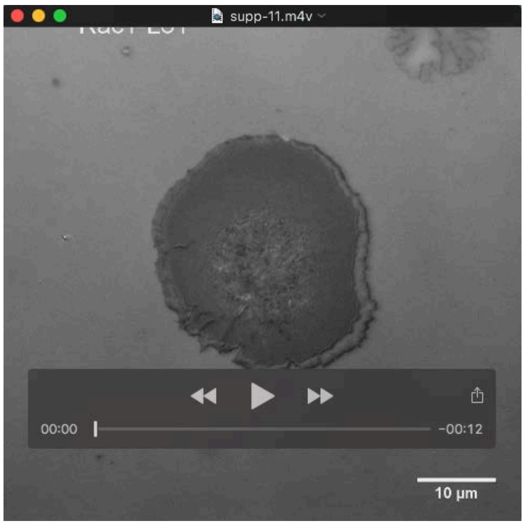

Movie 10: Spreading of cells expressing constitutively active Rac1 on galectin-8 coated substrates. Representative time-lapse series showing GFP-Rac1-L61 expressing cells on spreading galectin-8-coated substrate, imaged by interference reflection microscopy (IRM). The time interval is 5 seconds. Scale bar: 10 µm. Timestamp: mm:ss, the display rate: 15 frames per second.

Journal of Cell Science • Supplementary information

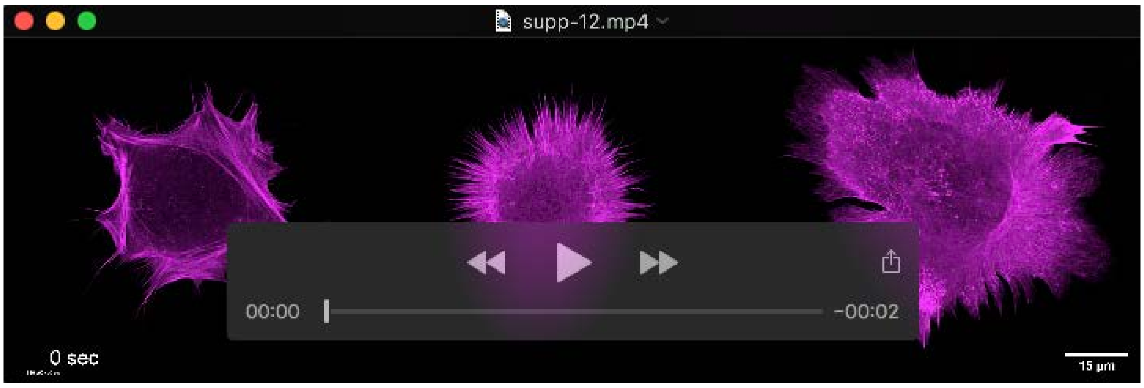

Movie 11: Spreading of cells on fibronectin (FN), galectin-8 (Gal) and a composite of fibronectin and galectin-8 (FN+Gal). Representative time-lapse series showing tdTomato-F-tractin expressing cells spread on fibronectin-, galectin-8-, or a combination of fibronectin and galectin-8- coated substrate four hours following plating, imaged by spinning disk confocal microscopy. The time interval is 30 seconds. Scale bar: 15 μm. The display rate: 7 frames per second.

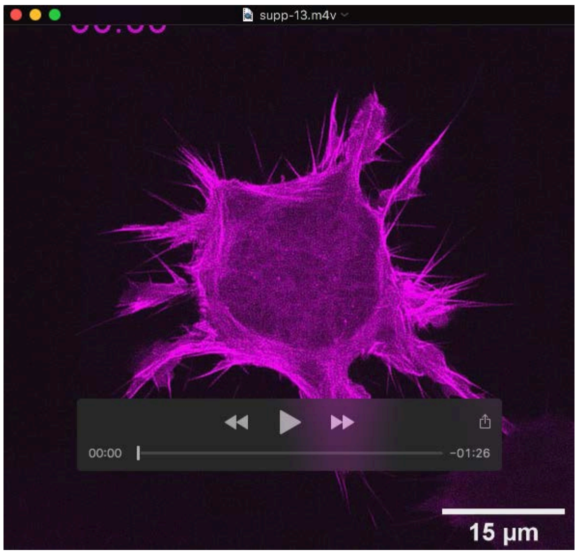

Movie 12: Spreading of cells on a composite of fibronectin and galectin-8 (FN+Gal). Representative time-lapse series showing tdTomato-F-tractin expressing cells spread on a combination of fibronectin and galectin-8 coated substrate, imaged by spinning disk confocal microscopy. The final concentration of both fibronectin and galectin-8 was 25 μg/ml. The film started at 30 minutes following cell plating. The time interval is 10 seconds. Scale bar: 15 μm. the display rate: 7 frames per second.

Journal of Cell Science • Supplementary information
