# Supplementary material for: Differential cellular responses to adhesive interactions with galectin-8- and fibronectin-coated substrates
Source: J Cell Sci. 2021 Apr 27;134(8):jcs252221. doi: 10.1242/jcs.252221 (PMC8106957; doi:10.1242/jcs.252221)
Supplement: Reviewer comments [file joces-134-252221_review_history.pdf]

Journal of Cell Science

Differential cellular responses to adhesive interactions with galectin-8- and fibronectin-coated substrates

Wenhong Li, Ana Sancho, Wen-Lu Chung, Yaron Vinik, Jurgen Groll, Yehiel Zick, Ohad Medalia, Alexander D. Bershadsky and Benjamin Geiger
DOI: 10.1242/jcs.252221

Editor: Arnoud Sonnenberg

Review timeline

Original submission:	25 July 2020
Editorial decision:	24 August 2020
First revision received:	6 January 2021
Editorial decision:	15 January 2021
Second revision received:	18 February 2021
Accepted:	3 March 2021

Original submission

<u>First decision letter</u>

MS ID#: JOCES/2020/252221

MS TITLE: Differential cellular responses to adhesive interactions with galectin-8 and fibronectin coated substrates

AUTHORS: Wenhong Li, Ana Sancho, Jurgen Groll, Yehiel Zick, Alexander D. Bershadsky, and Benjamin Geiger
ARTICLE TYPE: Research Article

We have now reached a decision on the above manuscript.

To see the reviewers' reports and a copy of this decision letter, please go to: https://submit-jcs.biologists.org and click on the 'Manuscripts with Decisions' queue in the Author Area. (Corresponding author only has access to reviews.)

As you will see, the reviewers gave favorable reports but raised some critical points that will require your attention. These concern the extent of integrin activation on the galectin 8 and fibronectin substrates and the effect of surface area of cells on the force measurements, the generality of your findings obtained with Hela-JW cells, the evidence that the cellular processes analyzed are true filopodia and not retraction fibers and the rationale for studying galectin 8 and fibronectin together. Furthermore, reviewer #3 expresses his/her concern about the effect of the outlier data points in Fig. 5b on the standard deviation.

We are aware that you may currently be unable to access the lab to undertake experimental revisions. If it would be helpful, we encourage you to contact us to discuss your revision in greater detail. Please send us a point-by-point response indicating where you are able to address concerns raised (either experimentally or by changes to the text) and where you will not be able to do so within the normal timeframe of a revision. We will then provide further guidance. Please also note that we are happy to extend revision timeframes as necessary.

© 2021. Published by The Company of Biologists under the terms of the Creative Commons Attribution License (https://creativecommons.org/licenses/by/4.0/).

If you think that you can deal satisfactorily with the criticisms on revision, I would be pleased to see a revised manuscript.

Please ensure that you clearly highlight all changes made in the revised manuscript. Please avoid using 'Tracked changes' in Word files as these are lost in PDF conversion.

I should be grateful if you would also provide a point-by-point response detailing how you have dealt with the points raised by the reviewers in the 'Response to Reviewers' box. Please attend to all of the reviewers' comments. If you do not agree with any of their criticisms or suggestions please explain clearly why this is so.

Reviewer 1

Advance summary and potential significance to field

This carefully performed study provides the first direct, comprehensive comparison of the responses of one particular cell line to adhesion to tissue culture substrates coated with galectin-8 compared to fibronectin. The authors present a number of new descriptive findings, including apparent differences in extent of cell spreading, nature of lamellipodial extension organization of the actin cytoskeleton, presence of focal adhesions and clusters of paxillin, roles of three major Rho GTPases, and overall strength of cell adhesion. These findings are novel albeit essentially descriptive and they provide a good foundation for considering that cell interactions with some extracellular molecules can be quite different from the classical studies of cell adhesion to fibronectin or collagen.

Comments for the author

This study is quite well documented experimentally with the application of a variety of high-end microscopy and AFM approaches. It will add to the descriptive foundation of knowledge about cell responses to different extracellular proteins and possible roles of Rho GTPases and the actin cytoskeleton. Although this study could be criticized for not providing major new mechanistic insights, this reviewer believes that it provides a useful, solid foundation for more detailed studies. Nevertheless, the authors need to rule out two obvious explanations for their differing findings for these two types of molecules implicated in cell adhesion.

1. An obvious explanation for many of the differences between the two molecules might be the extent of integrin activation, particularly with respect to the presence of focal adhesions and the cytoskeletal structures formed. Unless there is some compelling reason that it cannot be done, the authors need to do the very simple experiment of staining for activated integrins on each of the substrates, since at least some of the effects on galectin-8 could be due merely to a lack of activation of integrins.
Although this experiment would not be completely definitive for various technical reasons, this seemingly very obvious experiment would provide valuable insight into the findings. What the authors find, even if complex would not affect this reviewer's view concerning acceptability (unless this experiment is not performed).
Although one might wish for a better characterization of the types of integrins or other cell surface molecules being bound to each of these two substrates, and a mild concern that these experiments were performed with only a single cancer cell line rather than a couple of cell types, this reviewer does not feel that such valuable information is absolutely essential for this otherwise quite carefully performed initial comparison of two different adhesion proteins.

2. The authors need to comment on whether their findings concerning the AFM differences in adhesive strength between fibronectin and galectin-8 are at least partially affected by the surface area of the cells. That is, cells that are further spread are more difficult to detach from a substrate, and some means of ruling out this relatively trivial contribution to adhesive strength should be addressed.

Less major points:

© 2021. Published by The Company of Biologists under the terms of the Creative Commons Attribution License (https://creativecommons.org/licenses/by/4.0/).

3. The authors should be commended for their very careful morphometric quantification presented in Figures 6, 7, and 8. Considering that high standard, it is puzzling that in Figure 1e the measurements are shown as error bars rather than box-and-whisker plots. Most importantly, it is not clear whether the apparent differences in projected cell area are actually statistically significant.

4. This reviewer could not find a description of how the statistical analyses were performed, which were hopefully done using ANOVA and an appropriate post-hoc test.

5. Many of the experiments were performed with only single inhibitors or activators, and the knockdown experiments seem to have used only a single pool of siRNAs rather than the usual two independent siRNAs or a cDNA rescue. Although these additional experimental steps would be needed for a definitive study, this reviewer only wishes to note these weaknesses and comment that they may not be needed for this type of initial descriptive study.

6. This reviewer would have wished for a more definitive set of conclusions arriving at some valuable concepts rather than the long Discussion that was knowledgeable but did not provide us with some interesting new insights into cell science. For example, the synergistic effects of these proteins seemed quite interesting, but there were no insights into mechanisms. Nevertheless this point is not a fatal flaw and would not impede acceptability for publication in this reviewer's mind.

Reviewer 2

Advance summary and potential significance to field

This paper describes effects on HeLa JW cells when attaching and spreading on surfaces coated with galectin-8 as compared to fibronectin, or mixtures of the two. Some very interesting differences are found. The system with surfaces coated molecules is highly artificial, especially for galectin-8, a soluble lectin, which one would not expect to be found coated on a surface in vivo. Nevertheless, it is a very common model of cell-matrix interaction, adding galectin-8 to the range of molecule tested, is worthwhile. A number of elegant techniques are used.

Main findings are that cells spread more on galectin-8 and in a differe3nt way, that focal adhesions are lost on galectin-3, that cells bind with higher force to gaectin-8, and that there are some distinct effects of activating or suppressing Rac1.

Comments for the author

The Abstract could be more specific regarding main findings as decribed abobve.

Some issues need to be clearer, however:
Firstly, it would have been useful if the pages were numbered, and even better with numbered lines also.

Page 7, line 4. The Hela-JW cells were selected bbased on better adhesion to plastic. Are some molecular differences known about them, relevant for the present adhesion studies?

Page 8 (with title page as no 1), line 6-3 from bottom, Fig. S8. The text indicates that in some experiments fibronectin was coated in the presence of galectin-8, but this is not shown in Fig. S8. How is it explained that, as is said, the presence of 25 ug/ml galectin-8 did not reduce coating of fibronectin very much. Are they coating different sites on the glass plate?, or is galectin-8 bound to already bound fibronectin?

Page 9, Par 2, line 6-8. Why was the TDG added to cells before seeding? Then it would be expected to inhibit other galectins on and in the cells, and probably also taken up by pinocytosis. If the intention was to inhibit galectin-8 on the plate, then should not the plate be preincubated? For RGD the receptor is on the cell, so there this procedure is more understandable. In this place it is also stated that 10 mM TDG was used, but in another place (Fig. 5), 20 mM TDG was used. Why the difference?

© 2021. Published by The Company of Biologists under the terms of the Creative Commons Attribution License (https://creativecommons.org/licenses/by/4.0/).

Regarding method and Fig. 4. The word kymograph is only used in figure legends. It needs to be explained better. What is on the X and Y-axis in panel c) and d)?

Page 15, line 12-14. It is nice to give credit to the old reference by Kaufman and Lawless, but there is no evidence that the lectin they talk aboyt is galectin-8, not even a galectin, although it is probably galectin-1. So references should be given to the affinity of each of the two galectin-8 CRDs for TDG.

Page 16, par 2, and Fig. 5b. It is very good that the authors shows each data point as a dot. But this also reveals that the conclusion on page 16 is shaky. Both TDG and RGD seem to decrease the force of cell binding to fibronectin to about the same extent, but because of one high (outlier?) data point for TDG, this difference does not become statistically significant. Without this data point, the data for TDG and RGD would look about the same. This highlights the important problem, that one shall not draw conclusions only based on what calculates to be statistically significant or not, but also the size of the difference and what the data distribution looks like. So the text ion Page 16 needs to be reworded. TDG might very well have inhibited other galectins, and thereby perhaps reduced the surface exposure of integrins?

Both the Introduction and especially the Discussion are very lengthy. They contain useful information on cell adhesion to surfaces like in the present system, but not much about the relevant properties of galectin-8. How do the authors picture galectin-8 working as a matrix molecule. Does one CRD bind other matrix molecules, and the other the cll surface? The N-CRD has the highest affinity for sialylated galactosides, and also the highest affinity for cell surfaces and glycoproteins in general in many reports. It also gave strongest spreading of cells (Fig. S2). The C-CRD does not bind sialylated galectosides, and in general binds weaker to glycoproteins than the N-CRD. But it also has a peptide-binding site. So what is the role of the C-CRD in these experiments?

Reviewer 3

Advance summary and potential significance to field

In this manuscript, Li et al. compare how cells spread on galectin-8 and fibronectin-coated substrates. They made several interesting observations including the fact that Galectin8 appear to induce the formation of filopodia. Once my main criticisms are addressed, I believe this paper would be suitable to be published in JCS.

Comments for the author

Main comments
1) It is currently difficult to assess the variability and robustness of the findings:
N numbers are missing for most of the experiments performed. Very importantly the authors should provide both the number of cells analyzed but also the number of biological repeats performed. In particular, it appears that some of the experiments have been performed only once (supplementary figure 3A and 3B). The authors use siRNA to study the contribution of key cytoskeletal proteins. However, no validations of the siRNA used are provided. Authors should provide representative western blots. siRNA data should also be rescued or at least validated using another siRNA to ensure that the observations made are not due to off-target effects.
The authors rely on the over-expression of constitutively active GTPases constructs. The constructs used appear to be untagged; the authors should provide an explanation of how the cells were selected in these experiments.
All the experiments have been done in HeLa cells. It would be essential to validate critical findings in another cell line to demonstrate their generalization.

2) From the video, the filopodia look very different when cells are spreading on FN or galactin8. Can filopodia adhesions form within galactin8 filopodia? Or is FN required to initiate filopodia stabilization? It would be useful to assess if talin and Vasp can localize to filopodia tips in this context.

© 2021. Published by The Company of Biologists under the terms of the Creative Commons Attribution License (https://creativecommons.org/licenses/by/4.0/).

3) It would also be useful to perform live-cell imaging experiments to look at the filopodia behavior when cells are plated on FN, Galectin8, or both (the 4h experiment). In the picture provided, the actin structure displayed look like retraction fibers. The live imaging experiment would be critical to demonstrate that they are filopodia.

4) It would be helpful if the authors would provide a rationale for why they study FN and Galactin8 together. Why picking Galectin8 over any other ECM molecule?

First revision

Author response to reviewers' comments

MS ID#: JOCES/2020/252221
Response to Reviewers

Reviewer 1:

Reviewer 1 Advance Summary and Potential Significance to Field: This carefully performed study provides the first direct, comprehensive comparison of the responses of one particular cell line to adhesion to tissue culture substrates coated with galectin-8 compared to fibronectin. The authors present a number of new descriptive findings, including apparent differences in extent of cell spreading, nature of lamellipodial extension, organization of the actin cytoskeleton, presence of focal adhesions and clusters of paxillin, roles of three major Rho GTPases, and overall strength of cell adhesion. These findings are novel albeit essentially descriptive, and they provide a good foundation for considering that cell interactions with some extracellular molecules can be quite different from the classical studies of cell adhesion to fibronectin or collagen.

Response: We are grateful to the reviewer for highlighting the novelty of this paper. We believe that the new information, included in the revised version of the paper add new insights into the mechanisms involved in the sensing of molecularly compound matrix.

Reviewer 1 Comments for the Author: This study is quite well documented experimentally with the application of a variety of high-end microscopy and AFM approaches. It will add to the descriptive foundation of knowledge about cell responses to different extracellular proteins and possible roles of Rho GTPases and the actin cytoskeleton. Although this study could be criticized for not providing major new mechanistic insights, this reviewer believes that it provides a useful, solid foundation for more detailed studies. Nevertheless, the authors need to rule out two obvious explanations for their differing findings for these two types of molecules implicated in cell adhesion.

1. An obvious explanation for many of the differences between the two molecules might be the extent of integrin activation, particularly with respect to the presence of focal adhesions and the cytoskeletal structures formed. Unless there is some compelling reason that it cannot be done, the authors need to do the very simple experiment of staining for activated integrins on each of the substrates, since at least some of the effects on galectin-8 could be due merely to a lack of activation of integrins. Although this experiment would not be completely definitive for various technical reasons, this seemingly very obvious experiment would provide valuable insight into the findings. What the authors find, even if complex, would not affect this reviewer's view concerning acceptability (unless this experiment is not performed).

Response: We agree with this reviewer that the nature of the paxillin-containing focal adhesion-like structures observed in cells spreading on galectin needs to be directly explored. We have followed this advice, and checked the presence of total and activated β1- integrins in HeLa cells spreading on fibronectin or galectin-8 by staining with P5D2 and HUST-21 antibodies respectively. We also examined the spreading on galectin-8 substrate of B16 melanoma cells, stably expressing GFP-β3-integrin. Surprisingly, we found that unlike cells adhering to fibronectin, in which paxillin was associated with integrins and located at the end of stress fibers, the paxillin-rich clusters in cells

© 2021. Published by The Company of Biologists under the terms of the Creative Commons Attribution License (https://creativecommons.org/licenses/by/4.0/).

adhering to galectin-8 were devoid of integrin and associated with non-stress-fiber F-actin structures. This finding clearly indicates that galectin-8 substrate does not support the formation of genuine focal adhesions and, concomitantly enhance spreading. This new information is particularly meaningful in view of results presented in a different section of the paper, namely the capacity of galectin/fibronectin mixture to enhance spreading while allowing the cells to develop stress fibers and focal adhesions. We show these new results in Fig. 4 and Fig S8 and refer to them in both the Results and Discussion sections (Page 8-9 Line 208-225, Page 16 Line 460-467).

Although one might wish for a better characterization of the types of integrins or other cell surface molecules being bound to each of these two substrates, and a mild concern that these experiments were performed with only a single cancer cell line rather than a couple of cell types, this reviewer does not feel that such valuable information is absolutely essential for this otherwise quite carefully performed initial comparison of two different adhesion proteins.

Response: We agree that extending our studies to additional cell types, beyond HeLa is desirable, and in the revised version, we included new data showing that various types of cell lines, including primary murine cardiac fibroblasts, human dermal fibroblasts (HDF), osteosarcoma (U2OS) cells, fibrosarcoma (HT1080), melanoma (B16), and rat embryo fibroblasts (REF-52), display enhanced spreading and markedly reduced focal adhesion formation on galectin-8 as compared to the same cells growing on fibronectin, comparable to the observations obtained with HeLa-JW cells. We've added a new supplementary figure (Fig S3) showing these results. Description of these new data is added to the Results section (Page 9 Line 226-235) and to the Materials and Methods section (Page 25).

2. The authors need to comment on whether their findings concerning the AFM differences in adhesive strength between fibronectin and galectin-8 are at least partially affected by the surface area of the cells. That is, cells that are further spread are more difficult to detach from a substrate, and some means of ruling out this relatively trivial contribution to adhesive strength should be addressed.

Response: The difference in adhesive strength between galectin-8 and fibronectin coated substrates is indeed partially attributable to the larger projected cell area on galectin-8, even at the very early time point after seeding (5 minutes). However, the adhesion strength, normalized per the extended cell-substrate interface, is still considerably higher on galectin-8 than on fibronectin (see Fig 5C). We are grateful to the reviewer for addressing this issue and we've discussed it, in greater detail, in the revised version (Page 10 Line 263-267, Page 18 Line 531-534).

Less major points:

3. The authors should be commended for their very careful morphometric quantification presented in Figures 6, 7, and 8. Considering that high standard, it is puzzling that in Figure 1e the measurements are shown as error bars rather than box-and-whisker plots. Most importantly, it is not clear whether the apparent differences in projected cell area are actually statistically significant.

Response: In the revised version, we changed Figure 1E. We decided to retain the original curve because we think it is important to show the spreading curve as a function of time. We changed the representation of the error bars from the standard deviation to standard error of mean, to show that the variability is small. Concerning the statistical significance of the difference in projected cell area between cells on galectin-8 and fibronectin coated substrates, we calculated the *p* values using two-tailed t-test in all experiments, where the projected cell area was measured (Fig 1, 8B).

4. This reviewer could not find a description of how the statistical analyses were performed, which were hopefully done using ANOVA and an appropriate post-hoc test.

Response: We used two-tailed t-test and non-parametric two-tailed Mann-Whitney test for statistical analysis of the results. The appropriate references are now included in Materials and Methods.

5. Many of the experiments were performed with only single inhibitors or activators, and the knockdown experiments seem to have used only a single pool of siRNAs rather than the usual two

© 2021. Published by The Company of Biologists under the terms of the Creative Commons Attribution License (https://creativecommons.org/licenses/by/4.0/).

independent siRNAs or a cDNA rescue. Although these additional experimental steps would be needed for a definitive study, this reviewer only wishes to note these weaknesses and comment that they may not be needed for this type of initial descriptive study.

Response: We are grateful to this reviewer for the understanding of time limitations and permission to omit some rigorous controls of possible off-target effects of the siRNA used. The justification for this is that last generation of Dharmacon smart pool siRNAs are properly checked by the manufacturer and they rarely produce off-target effects. Nevertheless, to still address the concerns of this reviewer, we performed several additional validation experiments supporting that the most important effects of siRNA knock-downs found in this study are not attributable to off-target effects of the siRNAs, and are validated, by having >2 effective individual reagents. The validated siRNA include Cdc42 and Rac1 as shown in Fig S6.

6. This reviewer would have wished for a more definitive set of conclusions arriving at some valuable concepts rather than the long Discussion that was knowledgeable but did not provide us with some interesting new insights into cell science. For example, the synergistic effects of these proteins seemed quite interesting, but there were no insights into mechanisms. Nevertheless, this point is not a fatal flaw and would not impede acceptability for publication in this reviewer's mind.

Response: We addressed this comment of the reviewer, in the new experimental data obtained throughout the revision of this paper, and presented in the revised paper. Basically, in a mixed matrix, galectin-8, dominates spreading, and fibronectin dominates focal adhesion formation. We also show that each of these proteins moderately suppresses the activity of the other, though the two surfaces highly synergize in stimulating filopodia extension. These findings are included in the appropriate sections of the Results and the Discussion (Page 14, Line 395-402, Page 19 Line 552-564).

Reviewer 2 Advance Summary and Potential Significance to Field:

This paper describes effects on HeLa JW cells when attaching and spreading on surfaces coated with galectin-8 as compared to fibronectin, or mixtures of the two. Some very interesting differences are found. The system with surfaces coated molecules is highly artificial, especially for galectin-8, a soluble lectin, which one would not expect to be found coated on a surface in vivo. Nevertheless, it is a very common model of cell-matrix interaction, adding galectin-8 to the range of molecule tested, is worthwhile. A number of elegant techniques are used.
Main findings are that cells spread more on galectin-8 and in a different way, that focal adhesions are lost on galectin-8, that cells bind with higher force to gaectin-8, and that there are some distinct effects of activating or suppressing Rac1.

Response: we are grateful to this reviewer for the positive evaluation of our results and the appreciation of our main findings.

Reviewer 2 Comments for the Author:

1. The Abstract could be more specific regarding main findings as described above.

Response: In the revised version, we condensed the abstract and highlight the main findings, including the new findings obtained in the revision.

2. Some issues need to be clearer, however: Firstly, it would have been useful if the pages were numbered, and even better with numbered lines also.

Response: We are grateful for the suggestion, and in the revised version, we have included the page and line number.

3. Page 7, line 4. The Hela-JW cells were selected based on better adhesion to plastic. Are some molecular differences known about them, relevant for the present adhesion studies?

Response: HeLa-JW cells were chosen for this study, due to their uniform and reproducible behavior

© 2021. Published by The Company of Biologists under the terms of the Creative Commons Attribution License (https://creativecommons.org/licenses/by/4.0/).

on different extracellular matrices, including fibronectin and galectin-8. That said, we added Supplemental figure (Fig. S3), showing comparable behavior of other cells, including primary murine cardiac fibroblast, human dermal fibroblast (HDF), osteosarcoma (U2OS), fibrosarcoma (HT1080), melanoma (B16), and rat embryo fibroblast (REF-52).
We've added a supplementary figure (Fig S3) showing these results. Description of these new data is added to the Results section (Page 9 Line 226-235) and to the Materials and Methods section (Page 25).

Page 8 (with title page as no 1), line 6-3 from bottom, Fig. S8. The text indicates that in some experiments fibronectin was coated in the presence of galectin-8, but this is not shown in Fig. S8. How is it explained that, as is said, the presence of 25 ug/ml galectin-8 did not reduce coating of fibronectin very much. Are they coating different sites on the glass plate, or is galectin-8 bound to already bound fibronectin?

Response: To address this question, we performed additional experiments, testing fibronectin adsorption in the presence of galectin-8. The new data are added to Fig S8E. We checked the absorption of fibronectin at various concentrations (0, 5, 10, 15, 20, 25 μg/ml), without the addition of galectin-8 or mixed with 15 μg/ml or 20 μg/ml of galectin-8. We found the addition of galectin-8 reduced fibronectin absorption, at low fibronectin concentrations (≤15 μg/ml). However, at high fibronectin concentrations (≥ 20 μg/ml), the addition of galectin-8 did not affect the fibronectin absorption significantly. We have now indicated this in the legend to Fig 8 and refer the reader to Fig S8E. This correction does not affect our conclusion concerning the behavior of cells on the composite substrates.

4. Page 9, Par 2, line 6-8. Why was the TDG added to cells before seeding? Then it would be expected to inhibit other galectins on and in the cells, and probably also taken up by pinocytosis. If the intention was to inhibit galectin-8 on the plate, then should not the plate be preincubated? For RGD the receptor is on the cell, so there this procedure is more understandable. In this place it is also stated that 10 mM TDG was used, but in another place (Fig. 5), 20 mM TDG was used. Why the difference?

Response: We agree with the reviewer that TDG in the medium can, in principle, inhibit other galectins and produce other non-specific effects in the cells. However, only pre-incubation of the galectin-8 coated substrates with TDG is not sufficient, because the TDG binding to galectin-8 is reversible. Indeed, high concentration of TDG added during the seeding inhibited the force required to detach cells from both galectin-8 and (to a smaller extent) fibronectin substrates after 5 minutes of spreading (Fig 5B). However, long term incubation with TDG did not affect the cell spreading and focal adhesion formation on fibronectin, but completely blocked cell spreading on galectin-8 (Fig. S4D, E), suggesting that the effect of TDG is specific. Two different concentrations of TDG were used in different experiments by historical reasons. Both of these concentrations are saturating.

5. Regarding method and Fig. 4. The word kymograph is only used in figure legends. It needs to be explained better. What is on the X and Y-axis in panel c) and d)?

Response: We appreciate the reviewer pointing this out. We added detailed description of kymograph building in the legend to the new Figure 5.

6. Page 15, line 12-14. It is nice to give credit to the old reference by Kaufman and Lawless, but there is no evidence that the lectin they talk about is galectin-8, not even a galectin, although it is probably galectin-1. So references should be given to the affinity of each of the two galectin-8 CRDs for TDG.

Response: The affinity (K_d) of galactin-8 (N-terminal domain) for TDG is 61 μM (Delaine et al, 2008, J. Med. Chem. (PMID: 19053747). The affinity of the C-terminal domain was not determined, but given its relative structural similarity to the N-terminal region and to other galectins, one can expect it to be around that of Gal-8N and Galectin-1 (K_d 78 μM). In any event, the TDG concentration used in our study (20 mM) exceeds by far these K_d values and should provide maximal inhibition. We have now included the reference to Delaine *et al*, 2008 into the paper.

© 2021. Published by The Company of Biologists under the terms of the Creative Commons Attribution License (https://creativecommons.org/licenses/by/4.0/).

7. Page 16, par 2, and Fig. 5b. It is very good that the authors shows each data point as a dot. But this also reveals that the conclusion on page 16 is shaky. Both TDG and RGD seem to decrease the force of cell binding to fibronectin to about the same extent, but because of one high (outlier?) data point for TDG, this difference does not become statistically significant. Without this data point, the data for TDG and RGD would look about the same. This highlights the important problem, that one shall not draw conclusions only based on what calculates to be statistically significant or not, but also the size of the difference and what the data distribution looks like. So the text ion Page 16 needs to be reworded. TDG might very well have inhibited other galectins, and thereby perhaps reduced the surface exposure of integrins?

Response: We agree with this comment and are grateful to the reviewer for drawing our attention on the interpretation to these data. We have now used more appropriate non- parametric two tailed Mann-Whitney test for calculating the *p* values for this experiment and indicated them in Figure 5B. We agree that 10 mM TDG indeed decreased the force required to detach cells from the fibronectin coated substrate 5 minutes following plating, and mentioned it in the text of the Results section (Page 10 Line 277-281). The exact mechanism of this effect is not clear. The more important result of this experiment is that TDG but not RGD peptide decreased spreading of cells on galectin-8 coated substrate.

8. Both the Introduction and especially the Discussion are very lengthy. They contain useful information on cell adhesion to surfaces like in the present system, but not much about the relevant properties of galectin-8. How do the authors picture galectin-8 working as a matrix molecule. Does one CRD bind other matrix molecules, and the other the cell surface? The N-CRD has the highest affinity for sialylated galactosides, and also the highest affinity for cell surfaces and glycoproteins in general in many reports. It also gave strongest spreading of cells (Fig. S2). The C-CRD does not bind sialylated galectosides, and in general binds weaker to glycoproteins than the N-CRD. But it also has a peptide-binding site. So what is role of the C-CRD in these experiments?

Response: The question about the differences between N-CRD and C-CRD functions and the mechanism of their synergistic effects is essentially beyond the scope of this study. We have now referred to the recent review paper discussing this issue (Cagnoni *et. al*, 2020). One guess concerning the function of C-CRD, in these experiments, is that the large amount of glycoproteins and glycolipids on the cell surface and the large amount of galectin-8 on the surface compensates for the low intrinsic affinity. Galectin-8 may also form lattice on the cell surface, which may potentially enhance cell spreading, and C-CRD may be important for the lattice formation. In addition, N-CRD and C-CRD may serve as a bridge that one binds to the galectin-8 lattice and the other binds to the cell surface.

Reviewer 3 Advance Summary and Potential Significance to Field:

In this manuscript, Li et al. compare how cells spread on galectin-8 and fibronectin-coated substrates. They made several interesting observations, including the fact that Galectin8 appear to induce the formation of filopodia. Once my main criticisms are addressed, I believe this paper would be suitable to be published in JCS.

Reviewer 3 Comments for the Author:

Main comments

1. It is currently difficult to assess the variability and robustness of the findings: N numbers are missing for most of the experiments performed. Very importantly, the authors should provide both the number of cells analyzed but also the number of biological repeats performed. In particular, it appears that some of the experiments have been performed only once (supplementary figure 3A and 3B)

Response: We appreciate the comments of this reviewer, and we've added the number of cells (N) to the legends to Figure 5-8.

The authors use siRNA to study the contribution of key cytoskeletal proteins. However, no validations of the siRNA used are provided. Authors should provide representative western blots.

© 2021. Published by The Company of Biologists under the terms of the Creative Commons Attribution License (https://creativecommons.org/licenses/by/4.0/).

siRNA data should also be rescued or at least validated using another siRNA to ensure that the observations made are not due to off-target effects.

Response: The justification for this is that last generation of Dharmacon smart pool siRNAs are properly checked by the manufacturer and they rarely produce off-target effects. Nevertheless, to still address the concerns of this reviewer, we performed several additional validation experiments supporting that the most important effects of siRNA knock-downs found in this study are not attributable to off-target effects of the siRNAs, and are validated, by having >2 effective individual reagents. The validated siRNA include Cdc42 and Rac1 as shown in Fig S6.

The authors rely on the over-expression of constitutively active GTPases constructs. The constructs used appear to be untagged; the authors should provide an explanation of how the cells were selected in these experiments.

Response: The cells used were transfected with GFP labelled constitutively active Rho GTPases. We have now mentioned this in the figure legends.

All the experiments have been done in HeLa cells. It would be essential to validate critical findings in another cell line to demonstrate their generalization.

Response: We agree that extending our studies to additional cell types, beyond HeLa is desirable, and in the revised version, we included new data showing that various types of cell lines, including primary murine cardiac fibroblasts, human dermal fibroblasts (HDF), osteosarcoma (U2OS) cells, fibrosarcoma (HT1080), melanoma (B16), and rat embryo fibroblasts (REF-52), display enhanced spreading and markedly reduced focal adhesion formation on galectin-8 as compared to the same cells growing on fibronectin, comparable to the observations obtained with HeLa JW cells. We've added a supplementary figure (Fig 3) showing these results. Description of these new data is added to the Results section (Page 9 Line 226-235) and to the Materials and Methods section (Page 25).

2. From the video, the filopodia look very different when cells are spreading on FN or galactin8.Can filopodia adhesions form within galactin8 filopodia? Or is FN required to initiate filopodia stabilization? It would be useful to assess if talin and Vasp can localize to filopodia tips in this context.

Response: We have now assessed the talin and VASP localization by transfecting cells with the relevant GPF tagged constructs and found that both proteins localize to the tip of filopodia. The results are shown in new Fig S2, S7. In addition, we have shown that endogenous integrin β1 and GFP-integrin-β3 are localized to the tips of the adherent filopodia on galectin-8 (new Fig 4). As emphasized above, these integrins are localized to focal adhesions formed on fibronectin, but not to the paxillin-enriched clusters, typical for these cells on galectin-8

3. It would also be useful to perform live-cell imaging experiments to look at the filopodia behavior when cells are plated on FN, Galectin8, or both (the 4h experiment). In the picture provided, the actin structure displayed look like retraction fibers. The live imaging experiment would be critical to demonstrate that they are filopodia.

Response: We performed live-cell imaging to clearly distinguish between filopodia and retraction fibers in cells spreading on combined substrates of fibronectin and galectin-8. We found that after four hours of spreading there are both retracting and extending protrusions, as suspected by the reviewer. To elucidate it further, we performed the new experiments, 30 minutes after plating, and carefully verified that all filopodia are indeed protruding. These experiments confirmed the synergy between galectin and fibronectin in stimulating filopodia formation. These data are presented in the new Movies 11 and 12. The quantification of the filopodia number and length at the 30 minutes time point are presented in Fig S8C, and the new text, describing these data is added on Page 14, Lines 395-397, 399-402.

4. It would be helpful if the authors would provide a rationale for why they study FN and Galactin-8 together. Why picking Galectin8 over any other ECM molecule?

Response: The choice of mixing galectin-8 and fibronectin is motivated by the fact that these

© 2021. Published by The Company of Biologists under the terms of the Creative Commons Attribution License (https://creativecommons.org/licenses/by/4.0/).

proteins greatly differ in their intrinsic effects on cell spreading and on focal adhesion formation, which raised an intriguing question, whether a mixed matrix will demonstrate a clear dominance of one of these components, a suppression of both spreading and focal adhesion formation, or a certain form of synergy that is unique for the galectin-fibronectin mixture. As shown, the two molecules indeed synergize in stimulating filopodia formation. In the revised version of the text, we extended the discussion of this question (Page 19 Line 552-565).

Second decision letter

MS ID#: JOCES/2020/252221

MS TITLE: Differential cellular responses to adhesive interactions with galectin-8 and fibronectin coated substrates

AUTHORS: Wenhong Li, Ana Sancho, Wen-Lu Chung, Yaron Vinik, Jurgen Groll, Yehiel Zick, Ohad Medalia, Alexander D. Bershadsky, and Benjamin Geiger
ARTICLE TYPE: Research Article

We have now reached a decision on the above manuscript.

To see the reviewers' reports and a copy of this decision letter, please go to: https://submit-jcs.biologists.org and click on the 'Manuscripts with Decisions' queue in the Author Area. (Corresponding author only has access to reviews.)

As you will see, reviewers #1 finds that you have satisfactorily addressed their comments and recommend publication. However, reviewer #2 and #3 still have some minor issues that you will need to address before submitting your final manuscript. These concern some technical descriptions, validation of siRNAs and biological repeats. Furthermore, reviewer #3 mentions that panel S5H is missing. I trust that you will be able to properly deal with them and look forward to receiving a further revision of your paper.

We are aware that you may be experiencing disruption to the normal running of your lab that makes experimental revisions challenging. If it would be helpful, we encourage you to contact us to discuss your revision in greater detail. Please send us a point-by-point response indicating where you are able to address concerns raised (either experimentally or by changes to the text) and where you will not be able to do so within the normal timeframe of a revision. We will then provide further guidance. Please also note that we are happy to extend revision timeframes as necessary.

Please ensure that you clearly highlight all changes made in the revised manuscript. Please avoid using 'Tracked changes' in Word files as these are lost in PDF conversion.

I should be grateful if you would also provide a point-by-point response detailing how you have dealt with the points raised by the reviewers in the 'Response to Reviewers' box. Please attend to all of the reviewers' comments. If you do not agree with any of their criticisms or suggestions please explain clearly why this is so.

Reviewer 1

Advance summary and potential significance to field

See summary in my previous review. The added data have made this study even more interesting and useful to the field.

© 2021. Published by The Company of Biologists under the terms of the Creative Commons Attribution License (https://creativecommons.org/licenses/by/4.0/).

Comments for the author

The authors have very carefully and conscientiously addressed all concerns.
I recommend that this fine manuscript now be accepted for publication.

Reviewer 2

Advance summary and potential significance to field

This paper describes effects on HeLa JW cells when attaching and spreading on surfaces coated with galectin-8 as compared to fibronectin, or mixtures of the two. Some very interesting differences are found. The system with surfaces coated molecules is highly artificial, especially for galectin-8, a soluble lectin, which one would not expect to be found coated on a surface in vivo. Nevertheless, it is a very common model of cell-matrix interaction, adding galectin-8 to the range of molecule tested, is worthwhile. A number of elegant techniques are used.
Main findings are that cells spread more on galectin-8 and in a different way, that focal adhesions are lost on galectin-8, that cells bind with higher force to gaectin-8, and that there are some distinct effects of activating or suppressing Rac1.
Allthough many issues relain unclaer, the very detailed study in this paper makes a very valuable contribution to the field of glycobiology as related to cell adhesion.

Comments for the author

The authors have responded well to most critique, except regarding the description of Kymograph. Their response does not make sense. There is no kymograph in Fig. 5 and no detailed description of it. There are only brief descriptions in Fig. 1, 3 and S1, but nothing under Methods. One can glean that in this special application in cell imaging strips are imaged and then put side by side representing different time points. But to a non-expert in this, it could be explained better. Moreover it should be told what imaging software was used for this; was it automatic or manual etc. How were strips to follow selected.

Reading comments by other reviewers it can be seen that details are missing in other places also. So, as a general point, the most important is to let the reader know precisely how the experiment was done, and the results, so reader have chance to make his/her own interpretation.

Reviewer 3

Advance summary and potential significance to field

In the revised manuscript Li et al. new pieces of data that support their conclusion.

Comments for the author

Unfortunately, the authors did not fully revised the manuscript, and several of my initial concern remains.

1. It is rather surprising that the authors do not provide validation for the siRNA used.

The explanation that the reagents are well-validated by the manufacturer is completely inadequate. While this may be the case (I strongly doubt it), these reagents have not been validated using the experimental conditions used here.
In addition, siRNA silencing efficiency varies considerably depending on the transfection reagents, the target, the cell lines, and the timing of the experiment.
As the data presented here cannot be interpreted whiteout knowing the efficiency of the silencing used, the authors should provide at the very least validation of these reagents.

2. The n numbers (numbers of cells analyzed) as well as the numbers of biological repeats are still missing from a large number of figure panels.

© 2021. Published by The Company of Biologists under the terms of the Creative Commons Attribution License (https://creativecommons.org/licenses/by/4.0/).

Missing panels include:

1D, 3E, 3F, 5 (all panels), 8, S1D, S3E, S4C, S4E, S5B, S5G, S7F, S8C, S8D, S8E

Several figure legend state that "N ≥20 cells were assessed under each experimental condition" Imaging 20-40 cells can be trivial and done in one biological repeat. Therefore indicating the number of biological repeats is essential to assess the robustness of the findings.

3. Experiments presented in supplementary figure 3A and 3B have clearly be done only once. These should be repeated or removed from the manuscript.

Minor:
the panel S5H does not appear to exists but is listed in the figure legend

---

Second revision

Author response to reviewers' comments

MS ID#: JOCES/2020/252221
Response to Reviewers

Reviewer 1 Advance Summary and Potential Significance to Field:
See summary in my previous review. The added data have made this study even more interesting and useful to the field.

Reviewer 1 Comments for the Author:
The authors have very carefully and conscientiously addressed all concerns. I recommend that this fine manuscript now be accepted for publication.

Response: We are grateful to the reviewer for the positive recommendation.

Reviewer 2 Advance Summary and Potential Significance to Field:
This paper describes effects on HeLa JW cells when attaching and spreading on surfaces coated with galectin-8 as compared to fibronectin, or mixtures of the two. Some very interesting differences are found. The system with surfaces coated molecules is highly artificial, especially for galectin-8, a soluble lectin, which one would not expect to be found coated on a surface in vivo. Nevertheless, it is a very common model of cell-matrix interaction, adding galectin-8 to the range of molecule tested, is worthwhile. A number of elegant techniques are used.
Main findings are that cells spread more on galectin-8 and in a different way that focal adhesions are lost on galectin-8, that cells bind with higher force to gaectin-8, and that there are some distinct effects of activating or suppressing Rac1.
Although many issues remain unclear, the very detailed study in this paper makes a very valuable contribution to the field of glycobiology as related to cell adhesion.

Reviewer 2 Comments for the Author:
The authors have responded well to most critique, except regarding the description of Kymograph. Their response does not make sense. There is no kymograph in Fig. 5 and no detailed description of it. There are only brief descriptions in Fig. 1, 3 and S1, but nothing under Methods. One can glean that in this special application in cell imaging strips are imaged and then put side by side representing different time points. But to a non-expert in this, it could be explained better. Moreover, it should be told what imaging software was used for this; was it automatic or manual etc. How were strips to follow selected.

Reading comments by other reviewers it can be seen that details are missing in other places also. So, as a general point, the most important is to let the reader know precisely how the experiment was done, and the results, so reader have chance to make his/her own interpretation.

© 2021. Published by The Company of Biologists under the terms of the Creative Commons Attribution License (https://creativecommons.org/licenses/by/4.0/).

Response: We thank the reviewer for pointing out these questions. We have added more detailed description in the Materials and Methods section, which is now highlighted in yellow (Line 895- 899). In particular, we included now the reference to the ImageJ montage function we used.

Reviewer 3 Advance Summary and Potential Significance to Field:
In the revised manuscript Li et al. new pieces of data that support their conclusion.

Reviewer 3 Comments for the Author:
Unfortunately, the authors did not fully revised the manuscript, and several of my initial concern remains.

1. It is rather surprising that the authors do not provide validation for the siRNA used. The explanation that the reagents are well-validated by the manufacturer is completely inadequate. While this may be the case (I strongly doubt it), these reagents have not been validated using the experimental conditions used here. In addition, siRNA silencing efficiency varies considerably depending on the transfection reagents, the target, the cell lines, and the timing of the experiment. As the data presented here cannot be interpreted whiteout knowing the efficiency of the silencing used, the authors should provide at the very least validation of these reagents.

Response: We are very grateful to the reviewer for this comment. We performed the required validation experiments, and present the results in the Supplementary Figure 7. The validation of data with knock-downs of Rac1 and Cdc42 were already presented in the previous version. We have now added validation data with knock-downs of mDia2 and Arp2.
As for the knock-down of FMNL2, we did not get conclusive validation of the effect of SMART pool siRNA (catalog no. M-031993-01-0005) by the expression of individual siRNAs (catalog no. D-031993-01, D-031993-02), and given that we have clear effects by another formin (mDia2) knock-down, we prefer to withdraw the data concerning FMNL2. It does not affect the main message of the paper.

2. The n numbers (numbers of cells analyzed) as well as the numbers of biological repeats are still missing from a large number of figure panels. Missing panels include: 1D, 3E, 3F, 5 (all panels), 8, S1D, S3E, S4C, S4E, S5B, S5G, S7F, S8C, S8D, S8E. Several figure legend state that "N ≥20 cells were assessed under each experimental condition" Imaging 20-40 cells can be trivial and done in one biological repeat. Therefore, indicating the number of biological repeats is essential to assess the robustness of the findings.

Response: We have added the number of biological repeats to each of the figures (highlighted in yellow in the revised version of the manuscript).

3. Experiments presented in supplementary figure 3A and 3B have clearly been done only once. These should be repeated or removed from the manuscript.

Response: Since these results are of confirmatory nature and are not critically important for our paper, we decided to withdraw the data from the manuscript.

Minor Comment:

The panel S5H does not appear to exists but is listed in the figure legend

Response: We thank the reviewer for noticing. Indeed, the figure does not exist and we have removed the reference to it from the text.

© 2021. Published by The Company of Biologists under the terms of the Creative Commons Attribution License (https://creativecommons.org/licenses/by/4.0/).

Third decision letter

MS ID#: JOCES/2020/252221

MS TITLE: Differential cellular responses to adhesive interactions with galectin-8 and fibronectin coated substrates

AUTHORS: Wenhong Li, Ana Sancho, Wen-Lu Chung, Yaron Vinik, Jurgen Groll, Yehiel Zick, Ohad Medalia, Alexander D. Bershadsky, and Benjamin Geiger
ARTICLE TYPE: Research Article

I am happy to tell you that your manuscript has been accepted for publication in Journal of Cell Science, pending standard ethics checks.

Reviewer 3

Comments for the author

The authors have now addressed my remaining comments.
My main concern is that the n number used to build the conclusions presented here are very small for such experiments. However, as they are now clearly indicated in the figure legend, the readers can now decide for themselves.

© 2021. Published by The Company of Biologists under the terms of the Creative Commons Attribution License (https://creativecommons.org/licenses/by/4.0/).
